# Transnational readings in the Trumpocene: Kim Stanley Robinson’s *New York 2140* and Chris Beckett’s *America City*

**DOI:** 10.12688/openreseurope.17107.1

**Published:** 2024-10-09

**Authors:** Dolores Resano

**Affiliations:** 1 English Studies, Universidad Complutense de Madrid, Madrid, Spain

**Keywords:** climate fiction, populism, Trumpism, precarity, Brexit

## Abstract

This article discusses two climate fiction novels—one British, one American—that were written in the runup to two major political events on either side of the Atlantic in 2016—the Brexit referendum and the election of Donald Trump to the US presidency—and considers how their focus on a future climate emergency serves as an apt reflection on the mutual reinforcements of neoliberalism, precarization, and populism. By looking at these two novels together through the lens of right-wing populism and notions such as the Capitalocene (Moore), the Trumpocene (Colebrook) and the “critical utopia” (Moylan), I consider how the future climate catastrophes that these novels imagine are equally likely “to be used as opportunities to advance and entrench socially regressive forms of politics and unsustainable trajectories […] as inspire forms of ‘disaster collectivism,’ where acts of community and solidarity flourish” ( Newell, 2020: 157). As novels that are deeply concerned with the politics of the present, I consider how Robinson’s and Beckett’s novels are inspired by different utopian inflections that lead to different outcomes but similar diagnoses: that the worst effects of climate change will not be averted because humanity seems bent on its current trajectory.

## Plain Language Summary

This article discusses two climate-fiction novels—Kim Stanley Robinson’s *New York 2140* and Chris Beckett’s *America City*—in the context of the social and political moment in which they were written and published. It argues that the 2016 Brexit referendum and the election of Donald Trump as US President that same year are important background elements—even if never explicitly mentioned in the novels—that help us to consider the interrelation between climate change denialism, precarization, and far-right populism.

## Introduction

On the morning of 24 June 2016, the day after the Brexit vote, my English neighbor came down to our shared garden, his hair disheveled and his look genuinely puzzled, and exclaimed: “What the [expletive] just happened….?” Four months later, in early November, a similar scene probably repeated itself across many households in the United States when Donald Trump upset the 2016 prognostications for the US presidential election with an unexpected—at least for those who were not *really* paying attention—victory over the Democratic candidate Hillary Clinton. The Brexit vote and Trump’s election were read, back in 2016 and the years that followed, as shocking events in a political environment where politicians of far-right leanings like Marine Le Pen in France and Viktor Orbán in Hungary were mostly dismissed as isolated instances or read as democratic oddities, rather than as part of a larger and developing trend throughout the Western liberal and social-democratic political scene. A few years later, the 2024 elections to the European Parliament have soundly confirmed that these far-right, populist, and anti-establishment affiliations can no longer be considered mere leanings or intimations, but fully-fledged political actors that are here to stay, at least for the near future.

In hindsight, one can affirm that mainstream media readings of the 2016 political landscape in the United States and elsewhere were failing (or refusing) to recognize harbingers like the Tea Party movement of 2009 or, for instance, to factor in the effects of that long-standing British tradition of Euroscepticism, or to heed the warning signs that numerous scholars were advancing in their publications as early as 1999. ^1^ Back in 2008, scholars like Daniele Albertazzi and Duncan McDonnell had already identified that there was enough right-wing populist discourse circulating for them to publish the volume *Twenty-First Century Populism*, where contributors tackled the various populisms existing in Austria, Switzerland, Italy, Germany, The Netherlands, Sweden, France, Great Britain, and the Republic of Ireland. Indeed, Italy’s experience with a populist style of political leadership can be traced back to at least the 1990s, when figures like Silvio Berlusconi held the office of Prime Minister under four different administrations (1994–1995; 2001–2006; 2008–2011). Berlusconi presented himself as an “outsider” who could take the establishment by storm under the power of media celebrity. A decade after Albertazzi and McDonnell’s volume, studies of populism and the proliferation of far-right populisms in liberal democracies like the United States and much of Western Europe have mushroomed. ^2^ Moreover, countries like Ireland, Portugal, and Spain—which until very recently seemed somewhat foreign to the trend—by now have their own populist, far-right, and xenophobic factions to contend with, some of them already sitting in houses of Congress.

We can, indeed, talk of a transnational movement of twenty-first-century right-wing populism, as the trend is also visible in the social democracies of Latin America and in South-East Asian countries like India. Jair Bolsonaro’s tenure in Brazil (2019–2022) seemed, for a moment, like an exception in the region, because it was soon offset by a series of left-wing electoral victories in Mexico, Argentina, Uruguay, Chile, Colombia, and Bolivia; a sort of resurgence of the so-called “pink tide” of the early 2010s. However, this left-wing trend has been duly followed by a right-wing reaction, most notably the election in late 2023 of the ultra-liberal and openly populist Javier Milei as President of Argentina—a self-proclaimed anarcho-capitalist who promises to use his proverbial chainsaw against the state and the “political caste” to defend “los Argentinos de bien.” ^3^ As one instance follows another, there is an increasing confirmation of a turn against liberalism (or an illiberal turn, as has been sufficiently argued) ^4^ and the consolidation of right-wing and far-right populisms as *the* global trend of the twenty-first century in the West. The same Gadsen flags that were paraded during the attack on the US Capitol on 6 January 2021 were also flown in Argentina’s presidential inauguration in December 2023, a foreign symbol that would have no place in this southern country if it were not for the existence of a transnational network of populist, right-wing, and anti-establishment projects feeding each other across the West. ^5^ The ideological and strategic coordination between these groups is well-documented (most infamously in Steve Bannon’s “Gladiator School” in a monastery outside of Rome) and there is also a certain level of mutual recognition among voters and supporters. A global movement, yet locally-specific and diverse, this social and political trend shares in their populist and affective appeals—especially, the oppositional logic of “the people” versus “the elites,” as well as varying degrees of emotional investment in nostalgic retrotopias (or rather, uchronias, as these perfect pasts never existed), which can usually be achieved only by electing strong, autocratic leaders, among other salient features.

Scholars and critics continue to make the case for the unavailability (and inadvisability) of a clear-cut definition of populism—even of what is implied by the term “democracy” that is allegedly in peril today—and it is not the aim of this article to delve into these disciplinary distinctions, especially when so many excellent studies in the fields of political science, sociology, and philosophy have offered insight into the history and the commonalities, divergences, and complexities of the term. And yet, I find it necessary to draw an essential distinction between left-wing and right-wing populisms—at least those operating today in countries that are still recognized as full democracies—, one that is often muddled by conservative or right-wing media operators on account of their shared communicative and political styles, as if they were equally “dangerous” or “radical.” ^6^ The ethical implications of illiberal and exclusionary types of far-right populism (reactionary, xenophobic, ultra-conservative, homophobic, anti-feminist, ethno-nationalist, nativist, or neo-fascist) and their (more or less revolutionary or merely progressive) left-wing counterparts in Western democracies are far and apart. It is not a matter, in my view, of form but of content and gradation, of restricting or expanding fundamental and civil rights. In any case, and without getting into the particulars which are not the object of this study, I join scholars of populism in affirming that populism can broadly be understood as a style, as a rhetorical structure, as a means to communicate—even as an emotional appeal—, to organize and to operate politically (what Laclau and Mouffe would consider a “strategy”), through which each political or grassroots project articulates their own ideological and/or political agendas. Scholars tend to agree that a populist appeal tends to include an antagonistic framework, where “the people” find themselves confronting an agent of oppression (“the elite,” foreigners, Wall Street, “the establishment,” the media, globalists, or George Soros and “the Jewish cabal,” etc.) and where “the people” tends to be presented as a homogenous group, which makes it both coherent and capacious and, paradoxically, exclusive (of Others it identifies as “outsiders” or “internal enemies”). This group also tends to be constructed as a victimized majority (even if figures may or may not add up; hence Ronald Reagan’s “silent majority”) who legitimately represent the general will.

In this sense, some liberal analysists have lamented, well after the fact, that perhaps the best chance that the US Democratic Party could have had of defeating Donald Trump in 2016 would have been by nominating the Democratic Socialist Bernie Sanders to run as their 2016 candidate because, in many ways, his grassroots political style was closer to the populist leanings and desires that were already in the air—albeit with very different political aspirations. But, as Christian Parenti aptly put it, the Democrats “spurned and tried to sabotage Bernie Sanders and his class message. Trump took the Bernie-style populism, emptied it of real class politics, reduced it to a jumble of affective associations, and used it to beat-up the smug liberals of the professional managerial class” ( Parenti, 2016). If the Democratic leadership failed to register that there was indeed a populist current underway, it was clearly identified by many fiction writers-turned-journalists who followed the presidential campaign. For example, Ben Fountain’s columns for *The Guardian*, later published and expanded in *Beautiful Country Burn Again* (2018) or Jared Yates Sexton’s reporting for numerous outlets, which later produced the book *The People Are Going to Rise Like the Waters upon Your Shore* (2017), are just two examples of fiction writers chronicling the runup to the 2016 election and being clear-eyed in their recognition of a relentless cultural malaise that would give rise to an unstoppable populist wave. ^7^

If mainstream media commentary lagged in 2016 in their identification and analysis of the trend—perhaps distracted by media ratings, flawed interpretations of polling, and dismissive coverage on the liberal side—, fiction writing on both sides of the Atlantic did not, as I explore in this article. Ever attuned to the social and political disturbances that affect our present—this everyday reality that is, after all, the subject of literature—short-story writers and novelists produced some early works that were prescient, nuanced, and able to grasp this “new” reality as it was shifting and changing. Notable examples include Ali Smith’s Seasonal Quartet novels ( *Autumn*, 2016; *Winter*, 2017; *Spring*, 2019; and *Summer*, 2020), often hailed as one of the first literary responses to the Brexit campaign and to the rise of twenty-first-century nationalist populism. On the American side, it is not precisely the category of “Trump fiction” that produced the most engaging examples, but works that, while not explicitly referencing Trump’s election, managed to offer incisive interrogations of the very notions of “America” and democracy itself at a time of paradigmatic political change. While certainly a matter of personal preference, it is nonetheless safe to say that novels like George Saunders’s *Lincoln in the Bardo* (2017) and Viet Thanh Nguyen’s *The Sympathizer* (2015) can be read as clear-eyed reflections on the present even if not explicitly engaging with “Trump’s America.”

In this essay I contribute to the examination of these transatlantic and transnational trends by looking at two novels—one British, one American—that were written and published contemporaneously with both Brexit and the Trump election—in fact, written before either took place—and that, tuned to the disturbances of a rising populist wave, put the political imagination at the center of their fictions—albeit with different imagined outcomes. Moreover, I will argue that because both novels take place in the near future, they are both necessarily set against the background of climate change and ecological catastrophe, something that I argue reinforces the “reality effect” that near-future science fiction seeks to convey. Chris Beckett’s *America City* and Kim Stanley Robinson’s *New York 2140*, both published in 2017, are peri-apocalyptic narratives set in a not-too-distant future (Beckett’s is set within the lifetime of any present child) in which the ravages of climate change—produced in the novels by science denialism and a neoliberal maximization of profit at all costs—have materialized in climate catastrophes that lead to, among other things, political upheavals. Both novels present perfectly plausible future scenarios: Robinson’s flooded New York City in the year 2140 is the other side of the coin of Beckett’s dried-up, desertified American interior (and flooded coastlines, now called the “Storm Coast”).

As noted, both novels can be examined as part of that very productive corpus of works that, especially since the 2010s, has come to be designated as “climate fiction” (or cli-fi for short), a “popular, provocative, and hotly debated linguistic portmanteau” ( Leikam & Leyda, 2017: 110) that nonetheless can be uncontroversially described as “climate-conscious works” that are characteristically concerned with anthropogenic climate change (whether in the present or the future). Although there are clear precursors in the twentieth century, this type of work has proliferated especially since 2015 (as well as the scholarly studies on them) when, as Ursula K. Heise suggests, the effects of climate change have become increasingly visible in the Global North ( Heise 2024, 133). In line with this assessment, both Beckett’s and Robinson’s plots are driven by the (future) climate catastrophes they describe and the effects these have on their respective societies, starting with altered patterns of internal migration all the way to intimations of dystopian scenarios in which humans battle for dwindling resources, including water and land.

Here I want to consider how, beyond offering very suggestive depictions of climate devastation and its material, very tangible effects—that is, the precarious conditions that humanity will live under—these novels’ sustained focus on the political speaks loudly to our present populist moment, with its foregrounding of discourses of (dis)enfranchisement, (in)hospitality, (un)belonging, and (anti)cosmopolitanism ( Shaw, 2021). While the focus on the political is not in and of itself unusual in climate fiction because the political (mis)management and the devastations caused by climate change are deeply intertwined, here I am interested in following John Masterson’s argument that “there is palpable and portentous entanglement between climate change denial, national political bluster, and very material effects and affects when it comes to imagining human displacement in the future” ( Masterson, 2020: 7).

It is a well-worn trope that speculative fiction, though set in the future, tends to offer insightful reflections on the present; my argument here is that the combination of both genres—SF and cli-fi—works towards the foregrounding of a heightened sense of precariousness that also offers very insightful political readings of our present political moment, beyond the obvious conclusion that climate change is not something that will happen in a distant future but is already happening. In other words, I propose thinking about the effects and affects of climate change described in these novels as the representation of an exacerbated state of precarity—whether climatic, political, socioeconomic, or all at once—that allows us to consider the mutual reinforcements of precarity and right-wing populisms in the twenty-first century, as Paul Apostolidis (2022) has recently argued. Likewise, such an examination can allow us to imagine a type of politics that, in getting to the roots of precarity (beyond its customary understanding in socioeconomic terms) can actually contest Trumpism and similar discourses, as Apostolidis suggests.

## Cli-fi, the Capitalocene, and the Trumpocene

In his foreword to the anthology of climate fiction *Everything Change* (2016), Kim Stanley Robinson describes climate fiction not just as a subgenre of science fiction but as increasingly becoming “near-future science fiction”—which is concerned with events in the coming decades, in recognizable, real settings, unlike classical works of science fiction that could traditionally be set in distant places or galaxies and futures. As Robinson suggests, the rapid pace of technological and societal developments has exacerbated the effects of climate change and made them visible in the present and, as a result, near-future science fiction, due to its customary “fidelity to the real,” has increasingly turned towards and incorporated climate fiction. In fact, argues Robinson, climate fiction “has become in effect the realism of our time” ( Robinson, 2016a: ix). In other words, if contemporary science fiction tends to favor familiar and relatable settings (rather than the distant and the fantastic), it is impossible to write about the near future (or the present) without some reference to the ongoing process of climate change—“change” being, as Margaret Atwood has argued, quite a bland term that fails to capture the urgency and the impact that it will have on “everything,” not just on climate (hence the title of the anthology, *Everything Change*).

In the two novels under discussion, this sense of urgency and “presentness” is reinforced, I argue, by their peri-apocalyptic settings: in both *America City* and *New York 2140* the catastrophic climate “event” has already taken place but, rather than a dystopian aftermath of devastated landscape, what we get to witness is how humanity is tenaciously marching on in the midst of evolving disaster, adapting and even profiting from the newly altered geographical and sociopolitical landscapes—very much like the present, one could argue—, although as the plots move forward a darker underside begins to emerge. As a result, there is no Cassandra character in these novels, “a common cli-fi type usually portrayed as a scientist warning an ignorant public and/or a corrupt politician in vain about the dangers of climate change” ( Leyda, 2018: 96). On the contrary, both novels are deeply invested in the politics of disaster management, on how the crisis can be and is capitalized for political (Beckett) and financial (Robinson) gain, and on who is left behind or who is “othered” in such a process: who are the “elites” that hold on to power, who are the dangerous “im/migrants” that deplete already-dwindling resources, who are the former allies that are now turned into globalist enemies, and so on.

It is in this sense that I here eschew the notion of the Anthropocene as the hermeneutic approach that can allow us to read and interpret these novels’ reflections on the catastrophic effects of climate change and consider instead notions such as the Capitalocene and the Trumpocene, as these might be more apt terms to examine the environmental and cultural crises that both novels portray. If the Anthropocene is an understanding of humanity as a geological agent, the notion of the Capitalocene ( Moore, 2016), displaces humanity from the center and considers capitalism and neoliberalism instead, not just as “agents” of socio-political economy but as “ecological regimes.” The term “ecology” here implies a way of seeing and of organizing nature in which capital functions as the pivot point of an ecosystem where every constitutive part and living organism—including humans—exists in a symbiotic relationship. An ecological lens on capital, or what Moore calls “capital as world-ecology” ( Moore, 2014), stresses precisely this inter-connectedness, and rejects any Cartesian, binary way of thinking that, according to Moore, is at the root of the problem, both intellectually and politically: By upholding abstract divisions such as “Nature” and “Society” or “body” versus “mind”—as if society could exist without or outside nature, or as if humans were something other than nature—Moore argues that it is in the interest of capital to obscure this inescapable inter-connectedness in pursuit of endless consumption, exploitation, and profit. In other words, rather than focusing on humanity as *the* geological agent, the notion of the Capitalocene as ecosystem rightly places capital as the pivot of both the system and the crisis, a world ecology of capital, power, and nature in which these three elements are ontologically intertwined, in what Moore calls “a matrix of human and extra-human nature premised on endless commodification” ( Moore, 2011a: 114). Without eliding human agency or absolving humans of responsibility, a focus on capitalism as a totalizing system draws attention to its organizing logic and, possibly, to its faultlines.

Additionally, scholars like Claire Colebrook (2019) have elaborated on the notion of the Trumpocene for our current era, following Graham Readfearn’s first use of the term in 2016. This focus on Trumpism stresses the increase of science denialism couched in populist rhetoric, conspiratorial thinking, and the rejection of expertise, a type of thinking that, crucially, also flourishes under conditions of liberated markets and finance. We could say, then, that the Trumpocene is perhaps the latest iteration of the Capitalocene at a time that is witnessing the reinvigoration of right-wing populisms in the twenty-first century, because beyond the dismissal of the evidence of anthropogenic climate change lies an assault on environmental protections and regulatory power meant to unbridle what Moore calls the “dialectic of plunder and productivity” ( Moore, 2011b: 43). Therefore, my approach to *America City* and *New York 2140* draws from Moore’s notion of neoliberal ecology, Colebrook’s arguments on the entanglements of Trumpism, science denialism, and the figurations of climate catastrophe, and Apostolidis’ work on the mutual reinforcements of precarity and populism, as they elucidate how both novels function as visions of a near-future where neoliberalism, populism, and unbridled technological developments continue to thrive and to devastate. The crux of *New York 2140* lies precisely in the coexistence of two seemingly contradictory conditions—financialized accumulation in the age of climate change—which, in turn and as Roberto Ortiz argues in his review of Robinson’s novel, articulates what is “arguably the most insurmountable contradiction of late capitalism”: that climate change mitigation generates industries and products that continue to cement the dominance of finance, while at the same time, finance dominance exacerbates the effects of climate change ( Ortiz, 2020: 264).

Despite this seemingly unresolvable dynamic, *New York 2140* is set firmly within the utopian literary tradition, and by this I do not mean the classical literary utopias—which typically imagine improved societies in the future or at the edges of the known world—but in the sense developed by Tom Moylan in his seminal work *Demand the Impossible*, where he tried to capture the creative and critical capabilities of the utopian imagination and of utopian agency amid the countercultural moment of the 1960s and 1970s in the United States. For Moylan, the task of the “critical utopia” is to negate the very reality it opposes:

The task of an oppositional utopian text is not to foreclose the agenda for the future in terms of a homogeneous revolutionary plan but rather to hold open the act of negating the present and to imagine any of several possible modes of adaptation to society and nature based generally upon principles of autonomy, mutual aid, and equality. ( Moylan, 2014: 26)

I argue that, in line with Robinson’s long-standing commitment to socialist ideas and the utopian bent in much of his fiction, *New York 2140* seeks to both negate the current neoliberal totality and to test the horizon of the possible: What if we could “push the button” and open the doors to a post-capitalist future? How would we get there? In a challenge to that famous quip often attributed to Fredric Jameson that “it is easier to imagine the end of the world than to imagine the end of capitalism” ( Jameson, 2003: 76), ^8^ Robinson’s novel imagines a climate catastrophe that leads not to the end of the world but to the onset of some possible socialist organization, even if the novel ends with a deep awareness of its contingency and of the constant effort that will be needed for any gains to be upheld. It is, as Chris Pak suggests, a contingent utopianism, “aware that the utopian space [his] narrative[s] seeks to establish can be overturned” ( Pak, 2019: 105).

In the case of Beckett’s *America City*, the prospects seem to be much darker, and instead of asking “what if,” the novel seems to be asking “the more provocative and poignant ‘what when?’” ( Jensen, 2017). When will the devastating effects of climate catastrophe be effectively used by populist leaders to gain power and pit people against each other, even to the point of leading countries to consider invading their neighbors so as to seize control of precious, still-fertile land further north? The novel forcefully argues for the power of storytelling—showing how political rhetoric can be put to use both for positive and nefarious ends—in a near future in which the United States can still make a turn for the better. Things are moving in the wrong direction, but change can still happen, unlike in the United Kingdom, which is offered as a sad counterpoint that has already fallen under the thrall of nativist and xenophobic populism: “Fortress Britain these days was a nasty, desperate place […] a sinking pirate ship” ( Beckett, 2017a: 7). ^9^ With Britain already lost in the wake of Brexit—note how the “glorious” imperial past is here reframed as mere piracy— *America City* is in many ways an examination of the limits of empathy ( Beckett, 2019), something that Beckett would take up again in his 2020 novel *Two Tribes*, which is set in a post-Brexit Britain in the year 2266, when the European Union no longer exists. Neither do cars, hot water, or democracy, for that matter. I suggest that by setting the action closer to our lifetimes, *America City* keeps open the possibility that such a political *dénouement* may be averted, with Britain presented as a sad reminder of what may lie ahead. What is never in question, however, is that the effects of climate change will follow their course.

In short, I suggest that both *America City* and *New York 2140*—as much of recent climate fictions—share in that singular mode that Pilar Andrade (2024) calls “lo imposible cierto,” drawing from Jean-Pierre Dupuy’s notion of “l’impossible certain,” which refers to those as-of-yet unrealized hypotheses—often considered “unthinkable”—that are nonetheless factually certain because they are inevitable. In other words, and as Dupuy suggests, the unthinkable (“l’impossible”) is certain to happen because there is enough factual evidence that proves it. Therefore, Jensen correctly identifies that the question is perhaps not “what if” but simply “when?” or, I’d like to suggest, even more urgently, “how soon?” Moreover, and as Steven Shaviro argues in his review of *America City*, Beckett (and Robinson) join other contemporary authors in “taking seriously the grim prospect that nothing will be done in the coming decades to avert climate catastrophe, despite our clear awareness of the dangers and of our own responsibility for them” ( Shaviro, 2018).

To a large extent, populist rhetoric plays a part in this sense of inevitability. On the one hand, climate activism or even climate awareness are often decried as a “privilege” that can only be afforded by well-off “liberal elites,” a product for the progressive, urban-dwelling individual, something that the precarized working classes and the poor cannot afford to engage with. Indeed, as Dominik Schmidt concedes, “populism and the global climate movement are perceived as enormously influential but antagonistic forces” ( Schmidt, 2020). ^10^ Additionally, and more creatively, campaigns for climate awareness and sustainable development are sometimes framed by the reactionary right as part of a larger globalist hoax (concocted, again, by an undefined “elite”). ^11^ On the other hand, mainstreaming of climate awareness and “greenwashing” at the corporate level have also contributed to voiding of content and urgency the message of many ecological demands. In other words, climate change denialism (or even neglect) is yet another important issue that is often weaponized as part of the oppositional logic of “the people” vs. “the elites.” ^12^

In opposition to such bleak prospects, we can indeed reclaim the “environmentalism of the poor,” ^13^ which refutes simplistic (and interested) representations of environmental awareness and activism as a plaything for the rich (even if it may be so on occasion). “The Poor”, writes Joan Martinez-Alier, are

the *majority* of humankind, those who occupy relatively little environmental space, who have managed sustainable agroforestal and agricultural systems, who make prudent use of carbon sinks and reservoirs, whose livelihoods are threatened by mines, oil wells, dams, deforestation and tree plantations to feed the increasing throughput of energy and materials of the economy within or outside their own countries. ( Martinez-Alier, 2002: 13; emphasis in the original)

It is poorer and less industrialized societies who often bear the brunt of resource exploitation and waste management (those “negative externalities” of economic growth) and, as a result, “the poor are often on the side of resource conservation and a clean environment, even when they themselves do not claim to be environmentalists” ( Martinez-Alier, 2002: viii). In what follows, I consider how Robinson’s novel depicts precisely the opposite in great detail: a blooming Capitalocene in which the uber-rich continue to prosper and the precarious barely manage to survive.

## Drowning and thriving in the Trumpocene

*New York 2140* is set, as the title announces, in a flooded Manhattan in the coming century, where the devastating effects of two past catastrophic events of sea level rise, called the First and the Second Pulse, have been followed by periods of famine, economic crisis, and people fleeing from coastal cities to the interior. In the year 2140 Denver, Colorado, is the new capital city of the United States, as Washington D.C. has become uninhabitable. These migratory flows have also meant the temporary loss of New York’s status as the capital of global finance, but in the opening of the novel the city is thriving again, even if Lower Manhattan remains permanently flooded and Midtown is an intertidal zone. The city has resurged as a bustling “SuperVenice, majestic, watery, superb” ( Robinson, 2017: 6), according to one of the narrators; the cover image of the book is a very plausible rendition of the city as described in Robinson’s multifocal narrative. The resurgence also means that the city is ripe for the next real estate boom. The flooded areas in Lower Manhattan and Midtown—which had been abandoned in the past, were then taken over by scavengers and squatters, and eventually rehabilitated by groups of citizens who established cooperatives and communal forms of living that include farms on the rooftops—are now ripe for gentrification and are being targeted by the vulture capitalists uptown, whose aggressive takeover bids also include harassing tenants and sabotaging buildings in order to expel them. In short, the city keeps happily marching on to a new cycle of bubble, bust, and bailout; nothing has changed even if everything has changed. The city embodies the triumph of the neoliberal Capitalocene—even beyond human agency—and has adapted with great flexibility to all the challenges brought about by extreme weather and sea-level rise: canals of polluted, oily water are navigated in water taxis and *vaporettos*, while skybridges connect the skyscrapers whose ground floors have been turned into boathouses. The cityscape is also teeming with wildlife, with oyster farms in the canals and an abundance of fish, fowl, beavers, muskrats, seals: there is animal life everywhere. In the rest of the country, many species have become extinct or are on the verge of extinction, but a process of rewilding—which doubles as a lucrative TV show—is underway, forcing the migration of buffalos and polar bears to new habitats.

There is an unmistakable irony in this upbeat description, as it is far from some imagined, recovered Arcadia: as the novel makes increasingly clear, the effort to restore the balance of the ecosystem has become wholly integrated with the flows of finance capital and is contingent on the availability of bankable or monetized solutions: as noted, the assisted migration program is also an extremely popular reality show, the de-extinction of mammoths is justified solely by commodification, as there is a need to lower the pressure on the demand for elephants’ ivory tusks; the Intertidal Property Pricing Index, which tracks sea level change and predicts extreme weather events like tsunamis and storm surges, is used by investors to value drowned assets and to buy derivatives on underwater mortgages. In short, the thrust behind New York in 2140 closely resembles Moore’s notion of capitalism as world-ecology, which stresses precisely how financialization has permeated every aspect of human life (for instance, how pensions, retirement, healthcare, even life insurance are not only financial products in themselves but they in turn generate new derivatives that are speculated on in the global market). But the novel is also reminiscent of what Neil Smith (2007) has called “nature as accumulation strategy”: the efforts to mitigate the effects of climate change, in the shape of environmental regulations and requirements, give rise to new industries like the selling and purchase of carbon credits, reforestation quotas, negative emissions, catastrophe bonds etc., which generate huge profits that are, in turn, also financialized. Smith’s notion draws attention to how the rise of these industries meant to remediate the effects of the climate crisis in fact keep the financial wheel and the ecological crisis spinning: you can buy reforestation or carbon credits and keep polluting or chopping down trees because it is more profitable to pay someone else in the Global South to plant the required trees for you. As Ortiz suggests, the neoliberal ecology relies on these “strategies of accumulation by dispossession” ( Ortiz, 2020: 267) and the practical demands that arise as a result of climate change actually enhance “the dominance of the financial sector over the overall system, as more opportunities for commodification through financialization are created by the climate crisis itself” ( Ortiz, 2020: 266). It is, as Ortiz calls it, a “deadly symbiosis” ( Ortiz, 2020: 266) between nature and urban-centered finance capital, one for which the novel offers a seemingly impossible, radical and revolutionary resolution.

Two of the novel’s main focalizers, Mutt and Jeff—who are also the namesakes of a long-standing American comic strip—understand better than most of the other characters that in the New York of 2140 everything is interconnected globally—Manuel Castells’ notion of the network society (1996) is an apt description here—, in such a tight and seamless way that “defectors can’t get outside it” ( Robinson, 2017: 6). Even more so, a type of managerial and technocratic state has successfully blunted any desire for political action on the part of the citizenship ( Swyngedouw, 2010). It is thus only by understanding this global technocratic network, and its absolute totality, that a glitch in the system can be found and resistance can be effected. The urban space of *New York 2140* becomes thus, through the perspective of Mutt and Jeff, a potentially legible text, a situational representation of global neoliberalism through which it is possible to read the neoliberal world-ecology and our place within it. Mutt and Jeff, coders and drifters who live on-and-off the grid, spend the entire novel trying to find a glitch that will bring the global financial system down. The perspectives of the novel’s seven narrators or focalizers converge in the final sections, when a devastating hurricane hits the city and, in its aftermath, a horizontal logic of resistance gains impulse: Citizens of the intertidal succeed in coordinating, first locally, then globally, a debtors’ revolt, as people stop paying rent, mortgages, loans, credit card debt, etc., causing the predatory financial sector to crash and banks to be nationalized, in a reverse movement of what happened in 2008 when these were bailed out by the Federal Reserve. By refusing to inject capital into the system, a sort of dual power is established, very much in the way Jameson envisions in his manifesto *An American Utopia* (2016): ^14^ The multinational capitalist dominance eventually withers away, deemed no longer necessary, against the already-existing networks of cooperative ownership of buildings, self-sufficiency and communal living in the flooded areas. ^15^

This type of utopian possibility is qualitatively different from the one Robinson imagines in the short story “Mutt and Jeff Push the Button,” which is Robinson’s contribution to Jameson’s *An American Utopia* and which shares these two characters with the novel. In the short story, Mutt and Jeff “push the button” that will “end capitalism” by hacking into the computers at the World Trade Organization and rewriting “the code,” which is the “set of stupid laws” ( Robinson, 2016b: 100) that rule global financial capital. In rewriting them, they introduce “the opposite of efficiency, which is to say, justice” ( Robinson, 2016b: 104); the rewritten laws include, among other things, Jameson’s idea of universal drafting. If the story’s “solution” is cheekily simple, Robinson’s novel does take up Jameson’s call to think in utopian terms, as well as to re-politicize the issue at hand, which means really thinking “how we get to a better system” (Robinson in O’Keeffe, 2020), how we get from here to there. Jameson has repeatedly argued that dystopia has become our preferred speculative mode precisely because we live in a world in which “we can no longer imagine the future” ( Jameson, 2016: 13); in contrast, both Jameson and Robinson have always argued for utopian writing and thinking as a way of “saying NO” to the here and now (or what Moylan referred to as “negating the present”) while also thinking about alternatives to a present which is not necessary but contingent; in this, we can envision the future as disruption.

If in the short story “Mutt and Jeff Push the Button,” the button magically rewrites or edits the fourteen laws of global financial capitalism, immediately changing social organization and economic-political relations, the ending of the novel contains, however, a warning against such magical solutions. And here I want to draw attention to the correlation between people’s desire for easy solutions, populism, and climate change. To a great extent, magical thinking is part of the appeal of populist rhetoric, which tends to offer uncomplicated and straightforward analyses of and solutions to what are otherwise complex, multi-layered and often intractable social, political and/or economic problems. In a similar way, the discourse of eco-efficiency ( Martínez-Alier, 2002), with its emphasis on economic growth—albeit not at any cost—offers the possibility of mitigating the so-called “negative externalities” of such growth through capital-based stopgaps. Wealthy corporations in the industrialized world can pay farmers in the Global South to plant trees as carbon offsets; passengers can pay a small fee to offset their carbon emissions and continue to fly cheaply and often needlessly (when more environmentally friendly alternatives do exist). In other words, magical solutions are not only easy but require less work, and they also save us from struggle or disappointment. But by not targeting the root cause of the problem, they are also ineffective to change the status quo. In a similar way, the simplistic “solutions” often offered by the reactionary right (e.g. Brexit’s “Take Back Control,” Donald Trump’s “Build the Wall,” Rishi Sunak’s “Stop the Boats”) will not, in essence, solve any of the problems that “the people” are facing.

Robinson’s novel seems to be acutely aware of this, and the ending reminds us of the constant struggle that will be needed to uphold any gains that might have been achieved. As one of the narrators notes,

there was no guarantee of permanence to anything they did, and the pushback was ferocious as always, because people are crazy and history never ends, and good is accomplished against *the immense black-hole gravity of greed and fear*. Every moment is a wicked struggle of political forces, so even as the intertidal emerges from the surf like Venus, capitalism will be flattening itself like the octopus it biomimics, sliding between the glass walls of law that try to keep it contained…. ( Robinson, 2017, 604; emphasis added).

## Utopia can wait

A somewhat different take is offered in Beckett’s *America City*, although the novel also hinges on the exploitation of “the calculus of dread and comfort” ( Beckett, 2017a: 93), in this case by populist political operators. In his blog entry “Utopia Can Wait” ( 2021) Beckett shares his skepticism about the demands of the “more radical wing of climate change activists”—an end to greenhouse gas emissions and an end to capitalism as its condition of possibility—because “there is absolutely no way that a completely new and fully functional political and economic system is going to be constructed in the next few years” ( Beckett, 2021). Beckett calls for less “radical heroics” and more “meticulous practical work […] on problems such as mass energy storage, affordable green fuels, and carbon neutral cement” ( Beckett, 2021). While this somehow seems to dismiss the amount of scientific and practical work that is indeed being carried out in those areas, or the urgency of those in more precarious positions that do need “radical action” to be taken right now, Beckett is perhaps correct in identifying that “the political and business headaches that come with them” ( Beckett, 2021) may be the biggest challenge.

In thinking about “where we may be headed,” Beckett constructs a near future that is clearly recognizable, reinforcing the links between near-future SF, climate fiction, and realism. Thus, he seems to concur with Robinson’s diagnosis that all near-future science fiction will probably be climate fiction. Beckett’s cli-fi world building is a clear example of how the “future” has become the present, as there is no “abstracted other place” ( Moylan, 2014: 6) but the here and now: the United States of the next few decades, which has undergone a civil war after a catastrophic climate event and is once again on a populist drift. Interestingly, the question of *how* to write about climate change in a way that is somehow useful is at the forefront of Beckett’s project, as he considers the spectrum between optimism and hope, between utopia and dystopia, and what may move readers to action: “Appallingly bleak scenarios probably just encourage fatalism, while heartwarming stories of people in the future rebuilding civilisation from scratch after a catastrophe can seem positively appealing” ( Beckett, 2012). Indeed, this is one of the key questions when considering utopia; as Mathias Thaler puts it, how can social dreaming be empowering without being escapist wishful thinking? ( Thaler, 2019: 607). But as Thaler suggests, hope and despair are not polar opposites but can work together in complex ways that make them politically relevant, and this is the task of what scholars, including Moylan, have defined as the “critical dystopia.” This narrative has a “dual function,” which is, on the one hand, to “determine where danger looms in the present” and to “gesture towards potential responses in the future” ( Thaler, 2019: 608). For Thaler, they key characteristic of critical dystopias is that they

pivot around a type of hope that remains sensitive to the catastrophic failures of the past and alert to the immense perils of the present, without, however, foreclosing the prospect of a less oppressive, less violent, and less unequal future. ( Thaler, 2019: 608)

It may be argued that, between the poles of hope and despair, Beckett’s narrative seems to be moving closer to the latter—especially because of how recognizable, and sometimes even prescient, his descriptions of technology, social media, and its associated “algorithmic outrage” are. Political upheaval is at the forefront of Beckett’s narrative, and while Robinson’s utopia leads to a possible socialist future, Beckett’s dystopia leads to Trumpism and Brexit. Although Beckett started working on the idea that would give rise to *America City* back in 2012 (a short story titled “Destiny”), when he got back to it in 2016 he did so “against a new backdrop of real political events: first the Brexit vote in the summer of 2016, and then the rise of Donald Trump” ( Beckett, 2017b). Beckett offers some insight into how his creative process unfolded:

Obviously the book is deeply influenced by my thoughts about why and how these things happened and what I felt I was learning about how politics work. I felt very challenged by the fact that the 2016 Presidential election was going on as I wrote the book. Here I was, writing about an American politician winning an election by appealing to the tribal instincts of voters, and meanwhile in real life… In fact, in some respects what was happening in reality was *stranger* than what was happening in my book. I’m biased of course, but if I compare Slaymaker and Trump, it’s Trump who seems more like a made-up character. So there was a while there when I felt like the real world was overtaking me on the inside lane. ( Beckett, 2017b; emphasis in the original)

A familiar feeling many writers have expressed since 2016. *America City* is set in the United States roughly a century from now when, as a result of ongoing climate change, storms and hurricanes hit the coast every year, while vast areas of the interior in the south have become desertified and have insufficient water. Farmlands are no longer viable in those areas and their impoverished towns and cities cause a steady stream of climate refugees to head north every year. This internal migration receives an increasingly hostile welcome up north, whose inhabitants disparagingly call these refugees “storm people” or “barreduras,” and some northern states begin to consider imposing internal border controls to keep them out. The xenophobia that is typically directed at the foreigner is now fair game within national borders. The southern people themselves know this: “It’s like we’re not Americans anymore […]. It’s like we’re Mexicans or Haitians or something” ( Beckett, 2017a: 36). As becomes clear, the xenophobic discourse is not really about ethnic, racial or even cultural concerns, but more crudely about economics. This, as Zachary Levenson (2017) convincingly argues, is a key manoeuvre of populist obfuscation, where class interests are disguised as genuine political, social and/or cultural anxieties. ^16^

In the midst of all of this, Holly Peacock, a British PR professional living in the United states, begins working for a charismatic US Senator, Stephen Slaymaker, a “ferocious American nationalist, [a] bloodstained warrior from America’s wars in the African Copper Belt, [a] self-made man who thought that just because he’d been able to claw his way up from nothing, then everyone else could too” ( Beckett, 2017a: 15). He is the proverbial outsider who makes it into politics, a man who built the biggest trucking company in the country out of humble origins by his effort alone. He also has a track record of denying anthropogenic climate change (which is simply the result of “God’s will” [ Beckett, 2017a: 28]). Slaymaker decides to run for President under an ambitious program (“Reconfigure America”) that is meant to move the American population northward and thus remedy the north-south divide that is, once again, tearing the country apart: the internal migration of the dispossessed clashes against the interests of those who will soon be precarious too, and in the background there is an allegedly progressive, professional and educated class who is so detached from these material realities that they are disparagingly called “delicados.” Empathy is dangerous when survival is at stake; as one of the narrators admits, “It’s dangerous to feel sorry for [the barreduras], for that might mean an obligation to help them” ( Beckett, 2017a: 92). It may also require acknowledging and addressing the root causes that drive them north. Slaymaker’s relocation program is not motivated by any sort of humanitarian empathy either, but by pure political opportunism ^17^ and economic dogmatism: The people in the interior and on the Storm Coast are “soaking up tax money,” living where they can’t be productive and are thus “a drain on the country’s resources” ( Beckett, 2017a: 23). These opinions are echoed in the online public sphere, which Holly checks incessantly and is very adept at manipulating: “Fuck the storm people. *Fuck* them. If they’re dumb enough to still live in those places, how is that our problem?” ( Beckett, 2017a: 33; emphasis in the original).

Holly’s job is to “sell” the migration program to the people, especially northern voters, who have to be convinced that welcoming millions of climate refugees from the south is in their best interest. She finds a way that involves digging into the oppositional logic of “us versus them,” but displacing it towards the exterior: The neighbor up north, Canada. The affective logics of populism that both Holly and Slaymaker understand so well are put to play “to get America angry” ( Beckett, 2017a: 188). The plan is to rile people up against Canada so as to justify a war: “a bit of hostility toward Canada may not be a bad thing. Americans need somewhere to direct their frustrations other than to one another” ( Beckett, 2017a: 189). Building on a sense of grievance, on Canada’s “greediness” and “lack of solidarity,” Slaymaker’s team organizes a mob (of northerners and “barreduras”) and buses them to the Alaskan border under the false promise that Canadian authorities will let them in. Eventually the mob overrides the Canadian border forces, shots are fired, and chaos ensues. The stage is set, and Slaymaker sends tanks over the Alaska border and “bomb[s] the Ministry of Defense in Ottawa, reducing it to rubble” ( Beckett, 2017a: 333). The war eventually comes to an end with Canada’s defeat and utter destruction ( Beckett, 2017a: 334) and the annexation of a territory called “Northland,” the capital city of which will be America City. Holly knows the importance of this development, as the new “pioneers” who will settle across the border—people who had been until recently “foreigners in their own country” ( Beckett, 2017a: 328)—“were people who’d felt humiliated and powerless, and finally they were being given some hope and something to be proud of” ( Beckett, 2017a: 199). As with all nostalgic retrotopias, the movement is built on the myths of the past—references to Lincoln, the Civil War, and the western frontier abound—, as that which gives meaning to and articulates a new sense of collective pride and worth.

By the end of the novel, Holly becomes disillusioned with how it has all turned out, as she can no longer justify Slaymaker’s populist agenda as something meant for the greater good. She is also fully aware that if nothing is done, climate change will continue to render land uninhabitable and pushing populations further north, until “there’s nowhere left to go and your precious America will finally be completely fucked, along with the rest of the world” ( Beckett, 2017a: 337). But, as the narrator recognizes, there is no political will to be summoned, no will to implement the necessary measures to avoid catastrophe. Shockingly, but perhaps not surprisingly, the novel ends with Slaymaker’s political assassination at the hands of a young Inuit, a new “foreigner” in his own land (that has now become Northland). ^18^

## Some concluding thoughts

In late July of 2024, a British-born young man went on a killing rampage at the seaside town of Southport, west of Liverpool in England. He stabbed and killed three young girls and injured a dozen others, including their teachers. With the motives for the attack still under investigation by the local police, far-right agitators were quick to capitalize on the heinous crime and launched a powerful disinformation campaign that claimed that the killer was a recent refugee and stirred a mob to two weeks of riots which extended to other cities:

In Rotherham, they tried to torch a Holiday Inn Express sheltering asylum seekers. In Sunderland, they ransacked a police station and burnt out a Citizens Advice Bureau. In Belfast, they destroyed local shops, including one set up by a Syrian refugee and another by an immigrant from the Middle East, and broke into homes. In Liverpool, Hull, and Plymouth, they injured police officers. In Leicester, they menaced neighbourhoods, with Muslim residents fearful of leaving their houses. ( Lucas, 2024)

The leader of the far-right Reform UK party, Nigel Farage, continued to excuse the violence and to stoke fear even after the circumstances of the attack had become clear: “Was this guy being monitored by the security services? […] I just wonder if the truth is being withheld from us” (Farage in Lucas, 2024). As a recent editorial for *The Guardian* put it, the mobilization and weaponization of mob violence by the British far-right relied on recognizable themes: “a distrust of those in power, a willingness to blame economic and social ills on outsiders, on migrants and, in particular, Muslims, and the willingness of the cynical and the shameless to use these situations for their own profit” ( The Editors, 2024). Importantly, the editors also called politicians and the press on their responsibility “to think about the language they use in relation to migrants and multiculturalism” ( The Editors, 2024). As has been sufficiently argued, the British Conservative party has contributed in recent years to make the fringe mainstream with its inflammatory rhetoric and culture war agenda and has left Britain ripe for this type of xenophobic, chauvinistic and populist reaction.

This is not, however, an isolated incident, nor a tactic circumscribed to Britain. An eerily similar incident took place in the Republic of Ireland in November 2023, when three children and a woman were stabbed outside a primary school in Dublin city center around noon. The perpetrator was a man in his 50s, on whose identity and nationality the Garda (local police) refused to comment. ^19^ Misinformation quickly spread through Whatsapp, Signal, and Telegram about the identity of the attacker as an “illegal immigrant” and about the children being dead. Around 6pm, mob violence by far-right and anti-immigration agitators escalated, which led to a night of riots that included torched buses, looted shops, over sixty Gardaí assaulted, and certain neighborhoods being cordoned off. The level of violence was unprecedented in Dublin’s modern era, according to the Garda. In Spain, just two weeks after the events in Britain, the deadly stabbing of an 11-year-old while he was playing football at a municipal pitch became the excuse for similar campaigns of misinformation and criminalization of foreigners, especially those who resided in a nearby center for migrant children. That the perpetrator was a Spanish national was irrelevant to those intent on spewing misinformation and anti-immigration rhetoric and to right-wing political parties that repeated it. When the family of the victim asked the public and the media not to make such an unwarranted association between the attack and those seeking asylum, they were mercilessly abused on social media by the very same people that, in theory, were only concerned about their safety.

While it is dangerous (and irresponsible) to explain away this type of anti-immigration violence as the product of socioeconomic concerns or cultural fears—and one must be clear-eyed about how xenophobes and far-right agitators weaponize such vulnerabilities—it is pertinent to ask how this discourse may be countered. The association between socioeconomic precarity, lack of opportunity, and far-right affiliations as transparent and inevitable can and must be refuted, especially because it selectively ignores the millions who do live in precarious socioeconomic conditions and do not turn into thugs or violent xenophobes. And that includes the vast majority of migrants who are routinely demonized. It also bypasses the fact that xenophobia, the turn towards illiberalism, and the rise of far-right populism are also visible in Scandinavian countries, which have historically had the highest coefficients of social equality ( Hayes, 2024). Notwithstanding all of the above, and importantly, Paul Apostolidis demonstrates how relentless socioeconomic precarization tends to block people “from developing the critical dispositions” and the time needed for exercising “democratic citizenship” ( Apostolidis, 2022: 114). A precaritized existence can pose real and material impediments to political literacy, which can in turn facilitate the appeal of xenophobic rhetoric even across lines of class, gender, and ethnicity: this is what explains, for Apostolidis, that racialized, migrant and/or gendered people can sign on to the white supremacist discourse of Trumpism, with no apparent contradiction ( Apostolidis, 2022: 116).

There seems to be a real pushback against the very idea of a multicultural society; and while we may consider migratory movements the self-evident result of a process of market liberalization and neoliberal globalization since the 1990s, it is hard not to argue that the next great migratory movements will occur as a result of climate change, if it they are not already. The two novels examined here invite us to imagine not why or when this will happen, but how. In the expectation of this certainty, how to develop a type of politics that can actually contest Trumpism and exclusionary types of xenophobic populism? As Peter Newell argues in *Global Green Politics*, “disasters and catastrophes are as likely to be used as opportunities to advance and entrench socially regressive forms of politics and unsustainable trajectories […] as inspire forms of ‘disaster collectivism’, where acts of community and solidarity flourish” ( Newell, 2020: 157). Both Beckett’s and Robinson’s novels invite us to tread the line between utopia and dystopia, between magical thinking and revolutionary alternatives, and to consider, above all, how rhetoric and storytelling can be used both for laudable and nefarious ends.

## Ethics and consent

Ethical approval and consent were not required

## Data Availability

No data are associated with this article.

## Footnotes

^1^ To name but a few well-established examples: Margaret Canovan, “Trust the People! Populism and the Two Faces of Democracy” ( *Political Studies*; 1999, 47: 2–16); Margaret Canovan, *The People* (Polity Press, 2005); Ernesto Laclau, *On Populist Reason* (Verso, 2005); Cas Mudde, “The Populist Zeitgeist” ( *Government and Opposition*; 2004; 39.4: 541–563); Slavoj Žižek, “Against the Populist Temptation,” ( *Critical Inquiry*; 2006, 32: 551–74).

^2^ Besides the already-mentioned volume by Albertazzi and McDonnell (Palgrave Macmillan, 2008), see the more recent Cas Mudde & Cristóbal Rovira Kaltwasser, *Populism: A Very Short Introduction* (Oxford UP, 2017); Chantal Mouffe, *For a Left Populism* (Verso, 2018); Heike Paul, Ursula Prutsch & Jürgen Gebhardt (eds), *The Comeback of Populism. Transatlantic Perspectives* (Universitätsverlag Winter Heidelberg, 2019); Duncan McDonnell & Annika Werner, *International Populism. The Radical Right in the European Parliament* (Hurst, 2019); Aurelien Mondon & Aaron Winter, *Reactionary Democracy. How Racism and the Populist Far Right Became Mainstream* (Verso, 2020); Steven Forti, *Extrema derecha 2.0. Qué es y cómo combatirla* (Siglo XXI, 2021).

^3^ This is a classic appeal to “the people” adapted to the local idiosyncrasy. A literal translation from Spanish would be misleading and offer no clarification (“Argentines who do good”? “Argentines of good breeding”? “Honest Argentines”?); the appeal is both willfully blurry and class specific. The lack of definition of the group (just as in the case of “the people”) helps to keep the appeal open for various people to identify with and feel themselves being interpellated. My interpretation of this phrase (in the context of it being used by Milei and considering his politics) is “Argentines who are orderly, hard-working, and deserving—i.e. not poor—, because they contribute to rather than being a drain on the state.”

^4^ See, for example, Barry R. Posen, “The Rise of Illiberal Hegemony” ( *Foreign Affairs*, 13 February 2018); ); José Luis Valdés-Ugalde, “Hegemonía no liberal” ( *Norteamérica*; 2019, 14.2: 101–120); Donald E. Pease, “Donald Trump’s Settler-Colonist State (Fantasy): A New Era of Illiberal Hegemony?” (in *Trump’s America*, ed. Liam Kennedy [Edinburgh UP, 2020]: 23–52); Michael J. Boyle, “America and the Illiberal Order After Trump” ( *Survival*; 2020, 62.6: 51–76); Donna M. Goldstein, Kristen Drybread (eds.), *Corruption and Illiberal Politics in the Trump Era* (Routledge, 2023); Steven Hahn, “The Deep, Tangled Roots of American Illiberalism” ( *The New York Times*, 4 May 2024).

^5^ The Gadsen flag originated in the American Revolutionary War of 1775 and bears the motto “Don’t Tread on Me,” which helps to explain its repurposing as a libertarian symbol in the United States: the denunciation by American colonists of their oppression by the imperial master (Great Britain) in the eighteenth century is turned into a libertarian rallying cry against the US federal government in the twentieth. Its popularity among certain right-wing populist groups outside the United States in recent years signals a certain level of mutual recognition and ideological coordination among voters and supporters of the new reactionary right, who distrust “the State” and all established institutions.

^6^ I am keeping the discussion within the bounds of the democratic play, without entering the complexities posed by long-standing authoritarian regimes (whether left-wing or right-wing) in operation today in Latin America, Asia, and Europe. The distinction I am making between right-wing, far-right populist options and their left-wing counterparts is solely on account of their ideological and rhetorical appeals. Say, for example, between France’s Le Front National (Marine Le Pen) and La France Insoumise (Jean-Luc Mélenchon). John B. Judis’s distinction between “left-wing economic populism” and “right-wing *cultural* populism” is perhaps useful here ( *The Populist Explosion: How the Great Recession Transformed American and European Politics* [Columbia Global Reports, 2016]).

^7^ I would contend that Fountain was particularly well-equipped for this type of reporting as he had brilliantly captured and represented the chauvinistic, warmongering and, to a certain extent, populist sloganeering of post-9/11 American culture in his 2012 novel *Billy Lynn’s Long Halftime Walk*.

^8^ See also Jameson, 2016: 3.

^9^ “And America was a pirate ship too, of course, but at least for a moment it was pretty much afloat” ( Beckett, 2017a: 7).

^10^ Schmidt challenges this view and demonstrates through discourse analysis that the new climate movement (as exemplified by the activism of Greta Thunberg) makes use of both a populist framework (here understood solely as rhetorical style, not as ideology) and a depoliticizing strategy (the post-political here understood as universalizing claims that assume the absence of conflict and dissent). Their argument draws largely from Erik Swyngedouw’s provocative notion of “post-political populism” in relation to the depoliticization of climate change activism. See Erik Swyngedouw, “Apocalypse Forever? Post-political Populism and the Spectre of Climate Change” ( *Theory, Culture & Society*, 2010; 27[2–3]: 213–232).

^11^ Indeed, this is one of the five characteristics that Gregers Andersen identifies in cli-fi: the motif of “the conspiracy”: “anthropogenic global warming does not just appear as an object for objective (apolitical) science to investigate, but also as a narrative construction and political instrument [...] this is an epistemological issue that again and again emerges in the climate fictions [...]. In these fictions conspiracy is exactly what brings the entangled and complicated relationship between science and politics, truth and ideology to light.” (Andersen G: *Climate Fiction and Cultural Analysis: A New Perspective on Life in the Anthropocene*. Routledge, 2019: 64).

^12^ Or, as Greta Thunberg would have it, “our generation” (i.e. the young) vs. the “world leaders” (i.e. politicians, the older generations, irresponsible adults).

^13^ The term was first suggested by the historian Alberto Flores Galindo in 1988.

^14^ *An American Utopia. Dual Power and the Universal Army*, edited by Slavoj Žižek (Verso, 2016), is a provocative manifesto by Fredric Jameson followed by ten (mostly dissenting) responses by renowned Marxist scholars, such as Jodi Dean, Agon Hamza, Alberto Toscano, Žižek, and Robinson himself. Jameson’s text can be best understood as a thought-experiment meant to address our inability to imagine an alternative to (neoliberal) capitalism; it proposes a military model of collectivity based on universal conscription to the army that, in exchange for four hours of work a day, would fill in those social spaces that the neoliberal State has neglected or abandoned (for example, free clothing, food, healthcare, and housing, full employment, guaranteed basic income, enough paid leisure time, all of them services that current armies already provide). This universal army would function as a dual power, that is to say, a parallel power structure that does not challenge the State but that, eventually, would cause the State to become redundant and cease to exist. This proposed utopian model of mandatory and universal conscription, while it may seem too coercive, is meant to work as a sort of “cognitive mapping,” reminding us of the many coercions we already endure as part of our current conditions of existence in a globalized neoliberal economy that is increasingly dependent on precarization. If anything, the benefit of Jameson’s model is that, rather than being concealed and thus perceived as natural—even inevitable—, as neoliberal conditions often are, the coercion intrinsic to the universal army would be visible and transparent.

^15^ I want to credit here an observation made by my colleague Andrea Ruthven at the University of the Balearic Islands and which I haven’t had the opportunity to develop: the importance of water as that which enables connections, relation-ability, and the possibility of forming and sustaining new communities.

^16^ Likewise, there is no contradiction in the leader of “the people” being a millionaire (such as Trump and Berlusconi), because the designated antagonists are not the wealthy classes but “the political establishment,” to which they claim not to belong.

^17^ “We should be looking at this weather problem as an opportunity,” says Slaymaker ( Beckett, 2017a: 27).

^18^ Not surprisingly because of the United States’s long history of political assassinations (all the way from Lincoln to the Kennedys, Martin Luther King, and Malcom X), of which the recent attempt on Donald Trump’s life at a campaign rally is the latest example.

^19^ Eventually (much later) it was disclosed that the perpetrator was a naturalized Irish citizen of Algerian origin, who had been living in Ireland for over twenty years. However, the hashtag used by far-right agitators was #Irelandisfull, which had emerged in direct reference to recent refugee arrivals (especially after Russia’s invasion of Ukraine in 2022) and a housing shortage that has become especially acute in the last five years, together with an unprecedented cost-of-living crisis.
