# Transnational readings in the Trumpocene: Kim Stanley Robinson’s *New York 2140* and Chris Beckett’s *America City*

**DOI:** 10.12688/openreseurope.17107.2

**Published:** 2025-03-24

**Authors:** Dolores Resano

**Affiliations:** 1 English Studies, Universidad Complutense de Madrid, Madrid, Spain

**Keywords:** climate fiction, populism, Trumpism, precarity, Brexit

## Abstract

This article discusses two climate fiction novels—one British, one American—that were written in the runup to two major political events on either side of the Atlantic in 2016—the Brexit referendum and the first election of Donald Trump to the US presidency—and considers how their focus on a future climate emergency serves as an apt reflection on the mutual reinforcements of neoliberalism, precarization, and far-right populism. By looking at these two novels together through the lens of the Capitalocene (Moore), the Trumpocene (Colebrook) and the “critical utopia” (Moylan), I consider how the future climate catastrophes that these novels imagine can also serve to highlight the deep political implications of an advancing sense of precariousness as a result of climate exposure. As Newell suggests, climate catastrophes are equally likely “to be used as opportunities to advance and entrench socially regressive forms of politics and unsustainable trajectories […] as inspire forms of ‘disaster collectivism,’ where acts of community and solidarity flourish” ( Newell, 2020: 157). As novels that are deeply concerned with the politics of the present, I consider how Robinson’s and Beckett’s novels are inspired by different utopian inflections that, nonetheless, lead to similar diagnoses: that the worst effects of climate change are inevitable because humanity seems bent on its current trajectory. In doing so they showcase the potential of near-future science fiction to make legible the immediate political, social and environmental implications of ongoing climate change.

## Plain Language Summary

This article discusses two climate-fiction novels—Kim Stanley Robinson’s *New York 2140* and Chris Beckett’s *America City*—in the context of the social and political moment in which they were written and published. It argues that the 2016 Brexit referendum and the election of Donald Trump as US President that same year are important background elements—even if never explicitly mentioned in the novels—that help us to consider the interrelation between climate change denialism, precarization, and far-right populism.

## Introduction

On the morning of 24 June 2016, the day after the Brexit vote, my English neighbor came down to our shared garden, his hair disheveled and his look genuinely puzzled, and exclaimed: “What the [expletive] just happened….?” Four months later, in early November, a similar scene probably repeated itself across many households in the United States when Donald Trump upset the 2016 prognostications for the US presidential election with an unexpected—at least for those who were not *really* paying attention—victory over the Democratic candidate Hillary Clinton. It is worth remembering that back in 2016 and the years that followed, the Brexit vote and Trump’s first election were read as shocking events in a political environment where politicians of far-right leanings like Marine Le Pen in France and Viktor Orbán in Hungary were mostly dismissed as isolated instances or read as democratic oddities, rather than as part of a larger and developing trend throughout the Western liberal and social-democratic political scene. A few years later, the 2024 elections to the European Parliament and to the US presidency have soundly confirmed that these far-right, populist, and anti-establishment affiliations can no longer be considered mere leanings or intimations, but fully-fledged political actors that are here to stay, at least for the near future. The paradigm of Western liberal democracy as *the* most desirable political system for Western societies has come under intense scrutiny with the resurgence of xenophobic and nationalist populisms that often include a desire for autocratic governance, and that also heralds the demise of the post-45 transatlantic arrangement. The Trumpian present represents a paradigm shift when, in Nancy Fraser’s recent reframing of the old Gramscian adage, “the old is dying and the new cannot be born” yet ( Fraser, 2019).

In hindsight, one can affirm that mainstream media readings of the 2016 political landscape in the United States and elsewhere were failing (or refusing) to recognize harbingers like the Tea Party movement of 2009 or, for instance, to factor in the effects of that long-standing British tradition of Euroscepticism, or to heed the warning signs that numerous scholars were advancing in their publications as early as 1999. ^1^ Back in 2008, scholars like Daniele Albertazzi and Duncan McDonnell had already identified that there was enough populist discourse circulating for them to publish the volume *Twenty-First Century Populism*, where contributors tackled the various right-wing populisms existing in Austria, Switzerland, Italy, Germany, The Netherlands, Sweden, France, Great Britain, and the Republic of Ireland. Indeed, Italy’s experience with a populist style of political leadership can be traced back to at least the 1990s, when figures like Silvio Berlusconi held the office of Prime Minister under four different administrations (1994–1995; 2001–2006; 2008–2011). Berlusconi presented himself as an “outsider” who could take the establishment by storm under the power of media celebrity. A decade after Albertazzi and McDonnell’s volume, studies of populism and the proliferation of far-right populisms in liberal democracies like the United States and much of Western Europe have mushroomed. ^2^ Moreover, countries like Ireland, Portugal, and Spain—which until very recently seemed somewhat foreign to the trend—by now have their own populist, far-right, and xenophobic factions to contend with, some of them already sitting in houses of Congress.

We can, indeed, talk of a transnational movement of twenty-first-century right-wing populism, as the trend is also visible in the social democracies of Latin America and in South-East Asian countries like India. Jair Bolsonaro’s tenure in Brazil (2019–2022) seemed, for a moment, like an exception in the region, because it was soon offset by a series of left-wing electoral victories in Mexico, Argentina, Uruguay, Chile, Colombia, and Bolivia; a sort of resurgence of the so-called “pink tide” of the early 2010s. However, this left-wing trend has been duly followed by a right-wing reaction, most notably the election in late 2023 of the ultra-liberal and openly populist Javier Milei as President of Argentina—a self-proclaimed anarcho-capitalist who promises to use his proverbial chainsaw against the state and the “political caste” to defend “los Argentinos de bien.” ^3^ As one instance follows another, there is an increasing confirmation of a turn against liberalism (or an illiberal turn, as has been sufficiently argued) ^4^ and the consolidation of right-wing and far-right populisms as *the* global trend of the twenty-first century in the West. The same Gadsen flags that were paraded during the attack on the US Capitol on 6 January 2021 were also flown in Argentina’s presidential inauguration in December 2023, a culturally foreign symbol that would have no place in this Latin American country if it were not for the existence of a transnational network of populist, right-wing, and anti-establishment projects feeding each other across the West. ^5^ The ideological and strategic coordination between these groups is well-documented (most infamously in Steve Bannon’s “Gladiator School” in a monastery outside of Rome and, most recently, in the US Conservative Political Action Conference held in Maryland in February 2025). A global movement, yet locally-specific and diverse, this social and political trend shares in their populist and affective appeals—especially, the oppositional logic of “the people” versus “the elites,” as well as varying degrees of emotional investment in nostalgic retrotopias (or rather, uchronias, as these perfect pasts never existed), which can usually be achieved only by electing strong, autocratic leaders, among other salient features.

If mainstream media commentary lagged in 2016 in their identification and analysis of the trend—perhaps distracted by media ratings, flawed interpretations of polling, and dismissive coverage on the liberal side—, fiction writing on both sides of the Atlantic did not, and this is what I want to explore in this article. Ever attuned to the social and political disturbances that affect our present—this everyday reality that is, after all, the subject of literature—short-story writers and novelists produced some early works that were prescient, nuanced, and able to make legible this “new” reality as it was unforlding and shifting. Notable examples include Ali Smith’s Seasonal Quartet novels ( *Autumn*, 2016; *Winter*, 2017; *Spring*, 2019; and *Summer*, 2020), often hailed as one of the first literary responses to the Brexit campaign and to the rise of twenty-first-century nationalist populism in the United Kingdom. On the American side, it is not precisely the category of “Trump fiction” that produced the most engaging examples, but works that, while not explicitly referencing Trump’s election, managed to offer incisive interrogations of the very notions of “America” and democracy itself at a time of paradigmatic political change. ^6^

In this essay I contribute to the examination of these transnational trends by looking at two novels—one British, one American—that were written and published contemporaneously with both Brexit and the first Trump election—in fact, written before either took place—and that, tuned to the disturbances of a rising populist wave amid the extension of precarity in a world headed to climate catastrophe, put the political imagination at the center of their fictions—albeit with different imagined outcomes. I will argue that because both novels take place in the near future, they are necessarily set against the background of climate change, something that reinforces the “reality effect” that near-future science fiction seeks to convey. Chris Beckett’s *America City* and Kim Stanley Robinson’s *New York 2140*, both published in 2017, are peri-apocalyptic narratives set in a not-too-distant future (Beckett’s is set within the lifetime of any present child) in which the ravages of climate change—produced in the novels by science denialism and a neoliberal maximization of profit at all costs—have materialized in environmental catastrophes that lead to, among other things, political upheavals. Both novels present perfectly plausible future scenarios: Robinson’s flooded New York City in the year 2140 is the other side of the coin of Beckett’s dried-up, desertified American interior (and flooded coastlines, now called the “Storm Coast”).

As noted, both novels can be examined as part of that very productive corpus of works that, especially since the 2010s, has come to be designated as “climate fiction” (or cli-fi for short, following Dan Bloom’s coinage), a “popular, provocative, and hotly debated linguistic portmanteau” ( Leikam & Leyda, 2017: 110) that nonetheless can be uncontroversially described as “climate-conscious works” that are characteristically concerned with anthropogenic climate change (whether in the present or the future). Although there are clear precursors in the twentieth century, this type of work has proliferated especially since 2015 (as well as the scholarly studies on them) when, as Ursula K. Heise suggests, the effects of climate change have become increasingly visible in the Global North ( Heise, 2024, 133). At the same time, and as Claire Colebrook has argued in an excellent critique of the corpus, most recent climate fictions produced in the West are bound by the trappings of Enlightenment thought and, as such, they tend to be post-apocalyptic: that is, unable to imagine a world where humans have ceased to exist ( Colebrook, 2019b). Indeed, the Western, Anglo-centered perspective of these novels elides those “other” devastations and possible futures (indigenous, black, non-Western, non-dominant), but my interest in Beckett’s and Robinson’s novels lies precisely in their being peri-apocalyptic near-future narratives that hit so close to the current crisis of the Western, liberal present, thus transmitting a sense of urgency, especially in the political realm. While their plots are driven by ongoing climate events and the effects these have on their respective societies—starting with altered weather patterns and internal migration all the way to intimations of dystopian scenarios in which humans (and nations) battle for dwindling resources, including water and land—what holds my attention in particular is how each novel imagines outcomes that also serve as reflections on political agency, on how an increased sense of precariousness (at every level) can lead (or not) to communal efforts to affect change. Whether those visions are utopian and uplifting or simply dystopian is a major difference between the two novels, which serves to illustrate what Peter Newell argues in *Global Green Politics* (2020): that climate catastrophes are equally likely “to be used as opportunities to advance and entrench socially regressive forms of politics and unsustainable trajectories […] as inspire forms of ‘disaster collectivism,’ where acts of community and solidarity flourish” ( Newell, 2020: 157). My main argument is that, beyond offering very compelling depictions of climate devastation and its material, very tangible effects, these novels’ sustained focus on the political contributes to making legible what is at stake for the West, socially and politically, in an unmitigated climate catastrophe, when the stakes are already so high in the present. While Robinson’s utopian bent imagines the end of a technocratic, late-capitalist regime that is its condition of possibility, Beckett’s novel underscores how climate emergency also translates into uneven and increased precarity, exposure, and vulnerability and thus speaks loudly to our present populist moment, with its foregrounding of discourses of (dis)enfranchisement, (in)hospitality, (un)belonging, and (anti)cosmopolitanism ( Shaw, 2021). While the focus on the political is not in and of itself unusual in climate fiction—because the political (mis)management and the devastations caused by climate change are deeply intertwined—I am interested in following John Masterson’s argument that “there is palpable and portentous entanglement between climate change denial, national political bluster, and very material effects and affects when it comes to imagining human displacement in the future” ( Masterson, 2020: 7).

It is a well-worn trope that speculative fiction, though set in alternative timelines, tends to offer insightful reflections on the present; what I propose here is to think about the effects and affects of climate change described in these novels as the representation of an exacerbated state of precarity—whether climatic, political, socioeconomic, ontological, or all at once—that also allows us to consider the mutual reinforcements of precarity and right-wing populisms in the twenty-first century, as Paul Apostolidis (2022) has observed in a different context. The usefulness of reading fictional narratives as a way of “testing out” various political, economic or climate theories has been well argued by scholars in political and social sciences ( Cooper, 2021; Death, 2022; Hatzisavvidou, 2024); I argue that by making legible how political operators and global finance can exploit a more-than-certain future climate catastrophe, these narratives can also contribute to envision a type of politics that can actually contest the discursive and affective persuasiveness of exclusionary populist politics, as Apostolidis suggests. Robinson and Beckett offer different outcomes but very plausible readings of the immediate future (or the present), and put forward a sort of affective and cognitive mapping of how we get from here (now) to there (whatever “there” may turn out to be).

## Cli-fi, the Capitalocene, and the Trumpocene

In his foreword to the anthology of climate fiction *Everything Change* (2016), Kim Stanley Robinson argues that climate fiction, a subgenre of science fiction, is increasingly becoming “near-future science fiction”—which is concerned with events in the coming decades in recognizable, real settings, unlike classical works of science fiction that could traditionally be set in distant places or galaxies and futures. As Robinson suggests, the rapid pace of technological and societal developments has exacerbated the effects of climate change to the point that it has made them already visible in the present and, as a result, near-future science fiction has increasingly turned towards and incorporated climate fiction, due to its customary “fidelity to the real.” Robinson even argues that climate fiction “has become in effect the realism of our time” ( Robinson, 2016a: ix). In other words, if contemporary science fiction tends to favor familiar and relatable settings (rather than the distant or the fantastic), it is impossible to write about the near future (or the present) without some reference to the ongoing process of climate change—“change” being, as Margaret Atwood has argued, quite a bland term that fails to capture the urgency and the impact that it will have on “everything,” not just on climate (hence the title of the anthology, *Everything Change*).

In the two novels under discussion, this sense of urgency and “presentness” is reinforced by their peri-apocalyptic settings: in both *America City* and *New York 2140* the catastrophic climate “event” has already taken place but, rather than a dystopian aftermath of devastated landscape, we get to witness how humanity is tenaciously marching on in the midst of evolving disaster, adapting and even profiting from the newly altered geographical and sociopolitical landscapes, although as the plots move forward a darker underside begins to emerge. As a result, there is no Cassandra character in these novels, “a common cli-fi type usually portrayed as a scientist warning an ignorant public and/or a corrupt politician in vain about the dangers of climate change” ( Leyda, 2018: 96). On the contrary, both novels are deeply invested in the politics of disaster management, on how the crisis can be and is capitalized for political (Beckett) and financial (Robinson) gain, and on who is left behind or who is “othered” in such a process: who are the “elites” that hold on to power, who are the “dangerous” (im)migrants that allegedly deplete already-dwindling resources, who are the former allies that are now turned into “globalist” enemies, and so on.

It is in this sense that I here eschew the notion of the Anthropocene as the hermeneutic approach that can allow us to read and interpret these novels’ reflections on the catastrophic effects of climate change and consider instead notions such as the Capitalocene and the Trumpocene, as these might be more apt terms to examine the environmental and cultural crises that both novels portray. If the Anthropocene is an understanding of humanity as a geological agent, the notion of the Capitalocene ( Moore, 2016), displaces humanity from the center and considers capitalism and neoliberalism instead, not just as “agents” of socio-political economy but as “ecological regimes.” The term “ecology” here implies a way of seeing and of organizing nature in which capital functions as the pivot point of an ecosystem where every constitutive part and living organism—including humans—exists in a symbiotic relationship. An ecological lens on capital, or what Moore calls “capital as world-ecology” ( Moore, 2014), stresses precisely this inter-connectedness, and rejects any Cartesian, binary way of thinking that, according to Moore, is at the root of the problem, both intellectually and politically: By upholding abstract divisions such as “Nature” and “Society” or “body” versus “mind”—as if society could exist without or outside nature, or as if humans were something other than nature—Moore argues that it is in the interest of capital to obscure this inescapable inter-connectedness in pursuit of endless consumption, exploitation, and profit. In other words, rather than focusing on humanity as *the* geological agent, the notion of the Capitalocene as ecosystem rightly places capital as the pivot of both the system and the crisis, a world ecology of capital, power, and nature in which these three elements are ontologically intertwined, in what Moore calls “a matrix of human and extra-human nature premised on endless commodification” ( Moore, 2011a: 114). Without eliding human agency or absolving humans of responsibility, a focus on capitalism as a totalizing system draws attention to its organizing logic and, possibly, to its faultlines.

Additionally, scholars like Colebrook (2019a) have elaborated on the notion of the Trumpocene for our current era, following Graham Readfearn’s first use of the term in 2016. The focus on Trumpism stresses the increase of science denialism couched in populist rhetoric, conspiratorial thinking, and the rejection of expertise, a type of thinking that, crucially, also flourishes under conditions of liberated markets and uber-financialization (or what some scholars—most famously, Yanis Varoufakis, among others—have designated as “technofeudalism”). We could say, then, that the Trumpocene is perhaps the latest iteration of the Capitalocene at a time that is witnessing the reinvigoration of reactionary populisms in the twenty-first century, because beyond the dismissal of the evidence of anthropogenic climate change lies an assault on environmental protections and regulatory power meant to unbridle what Moore calls the “dialectic of plunder and productivity” ( Moore, 2011b: 43). ^7^ Therefore, my approach to *America City* and *New York 2140* draws from Moore’s notion of neoliberal ecology, Colebrook’s arguments on the entanglements of Trumpism, science denialism, and the figurations of climate catastrophe, and Apostolidis’ work on the mutual reinforcements of precarity and populism, as they elucidate how both novels function as visions of a near-future where neoliberalism and unbridled technological developments continue to thrive and to devastate. The crux of *New York 2140* lies precisely in the coexistence of two seemingly contradictory conditions—financialized accumulation in the age of climate change—which, in turn and as Roberto Ortiz argues in his review of Robinson’s novel, articulates what is “arguably the most insurmountable contradiction of late capitalism”: that climate change mitigation generates industries and products that continue to cement the dominance of finance, while at the same time, finance dominance exacerbates the effects of climate change ( Ortiz, 2020: 264).

Despite this seemingly unresolvable dynamic, *New York 2140* is set firmly within the utopian literary tradition, and by this I do not mean the classical literary eutopias—which typically imagine improved societies in the future or at the edges of the known world—but in the sense developed by Tom Moylan in his seminal work *Demand the Impossible*, where he tried to capture the creative and critical capabilities of the utopian imagination and of utopian agency amid the countercultural moment of the 1960s and 1970s in the United States. For Moylan, the task of the “critical utopia” is to negate the very reality it opposes:

The task of an oppositional utopian text is not to foreclose the agenda for the future in terms of a homogeneous revolutionary plan but rather to hold open the act of negating the present and to imagine any of several possible modes of adaptation to society and nature based generally upon principles of autonomy, mutual aid, and equality. ( Moylan, 2014: 26)

I argue that, in line with Robinson’s long-standing commitment to socialist ideas and the utopian bent in much of his fiction, *New York 2140* seeks to both negate the current neoliberal, financialized totality and to test the horizon of the possible: What if we could “push the button” and open the doors to a post-capitalist future? How would we get there? In a challenge to that famous quip often attributed to Fredric Jameson that “it is easier to imagine the end of the world than to imagine the end of capitalism” ( Jameson, 2003: 76), ^8^ Robinson’s novel imagines a climate catastrophe that leads not to the end of the world *per se* but to the withering of the capitalist order after a collective and revolutionary act. As Colebrook has noted in her critique of the Western cli-fi canon, “the ‘end of the world’ has come to mean the end of ‘our’ way of life” ( Colebrook, 2019b: 264), of the “capitalist, imperialist, hyper-consuming, enslaving” (i.e. Western) part of humanity, and it is indeed unthinkable and radical to imagine an end “that would not be an end for us, and that might generate another world” ( Colebrook, 2019b: 264), one not necessarily tied to capitalist modes of production and urbanity. In this sense, the novel is open-ended and concludes with a deep awareness of its contingency and of the constant effort that will be needed for any gains to be upheld. It is, as Chris Pak suggests, a contingent utopianism, “aware that the utopian space [his] narrative[s] seeks to establish can be overturned” ( Pak, 2019: 105). While the post-capitalist outcome at the end of the novel, when all the different narrators/focalizers converge, suggests some sort of collective, coordinated action, Colebrook is skeptical and argues instead that this coming together might be nothing more than an intersection of “individual projects and lives” that does not fundamentally challenge “that same ongoing spirit of individualism, progress, and fortune” that has characterized Western liberalism since the Enlightenment period ( Colebrook, 2019b: 271). This is no doubt an excellent point, but I intuit that even via Robinson’s “Marxist finance analytics” ( Colebrook, 2019b: 272) and despite the somewhat fortuitous revolution at the end, the novel contributes to making legible what is at stake in a world where every aspect of life has been financialized, when climate challenges become opportunities for profit, and when corporate, technological giants have almost absolute control over nature (including humans) though endless streams of data. The acceleration of the Capitalocene seems to offer nothing more than the same old class exploitation in even worse, regressive form: the choice seems to be between “precarity capitalism” ( Azmanova, 2020), “neofeudalism” ( Dean, 2020), or “technofeudalism” ( Varoufakis, 2023). Technological advances, rather than being used to alleviate the living, material conditions of the most precarious and of a distressed environment, become the facilitators of even more precarity, exploitation, and destruction, even for those living at the very center of the system (New York) that has wreaked so much havoc in the first place.

In the case of Beckett’s *America City*, the prospects seem to be even darker, and instead of asking “what if X happened,” the novel seems to be asking “the more provocative and poignant ‘what when?’” ( Jensen, 2017). When will the devastating effects of climate catastrophe be effectively used by populist leaders to gain power and pit people against each other, even to the point of leading countries to invade their neighbors so as to seize control of precious, still-fertile land further north? The novel forcefully argues for the power of storytelling—showing how political rhetoric can be put to use both for positive and nefarious ends—in a near future in which the United States can (maybe) still make a turn for the better. Things are moving in the wrong direction, but change can still happen, unlike in the United Kingdom, which is offered as a sad counterpoint that has already fallen under the thrall of nativist and xenophobic populism: “Fortress Britain these days was a nasty, desperate place […] a sinking pirate ship” ( Beckett, 2017a: 7). ^9^ With Britain already lost in the wake of Brexit—note how the “glorious” imperial past is here reframed as mere piracy— *America City* is in many ways an examination of the limits of empathy ( Beckett, 2019), something that Beckett would take up again in his 2020 novel *Two Tribes*, which is set in a post-Brexit Britain in the year 2266, when the European Union no longer exists. Neither do cars, hot water, or democracy, for that matter. I suggest that by setting the action closer to our lifetimes, *America City* keeps open the possibility that such a political *dénouement* may be averted, with Britain presented as a sad reminder of what may lie ahead. What is never in question, however, is that the effects of climate change—and humanity’s lack of action—will follow their course. Therefore, if Jensen correctly identifies that the question is perhaps not “what if” but simply “when?”, I would suggest that it is the even more urgent “how soon?” As Steven Shaviro argues in his review of *America City*, Beckett (and I would add Robinson too) join other contemporary authors in “taking seriously the grim prospect that nothing will be done in the coming decades to avert climate catastrophe, despite our clear awareness of the dangers and of our own responsibility for them” ( Shaviro, 2018). As Lawrence suggests, while the “dystopian actuality” ( Lawrence, 2020: 307) is already visible in many parts of the Global South, it seems that the climate fictions of the Global North have been anxiously grappling with this certainty for a while now.

There is nothing inherently defeatist in this sense of inevitability; rather, I contend that both *America City* and *New York 2140*—as much of recent climate fictions—share in that singular mode that Pilar Andrade (2024) calls “lo imposible cierto,” drawing from Jean-Pierre Dupuy’s notion of “l’impossible certain” (2002), which refers to those as-of-yet unrealized hypotheses—often considered “unthinkable”—that are nonetheless *factually certain* because they are inevitable. The unthinkable (“l’impossible”) is certain to happen because there is enough factual evidence that proves it. However, in this scenario of factual inevitability, it is relevant to consider what level of agency is left for humans. As Nick Lawrence convincingly argues—drawing on Sasha Lilley’s work—, catastrophist rhetoric can often lead to not just “apocalypse fatigue” ( Lawrence, 2020: 307) but to responses—both on the left and on the right—that elide any human agency in bringing about change. On the one hand, a belief that the collapse of “the system” will bring about revolutionary change by itself, which absolves humans from the responsibility to act. On the other, what Lawerence calls a “voluntarist” response, that “heralds the acceleration of economic disaster, climate change and intensifying state oppression as the necessary, indeed only, conditions enabling the prospect of revolutionary transformation” ( Lawrence, 2020: 308). Both responses—that the system has reached its limit or that it is best to burn it all down—mask an underlying, deep sense of despair, according to Lawrence, and should be resisted.

Additionally, the contemporary reactionary right often weaponizes the call to action under the guise of, alternatively, denialism, conspiracy, or simply the oppositional logic of populism. This is one of the interrelated aspects that Colebrook (2019a) identifies in the Trumpocene, how climate activism or even climate awareness is decried as a “privilege” that can only be afforded by well-off “liberal elites,” a product for the progressive, urban-dwelling individual, something that the precarized working classes and the poor cannot afford to engage with, a successful framing of the global climate movement and “the people” as antagonistic forces ( Schmidt, 2020). ^10^ Other times, and more creatively, campaigns for climate awareness and sustainable development are framed by the reactionary, nationalist right as part of a globalist hoax (concocted, again, by an undefined “elite” and scheming, supra-national entities like the UN or the EU). Non-ironically, Donald Trump’s “drill, baby, drill” tagline also means de-regulating any environmental protections that at present impede wealthy corporations from the unrestricted freedom to pollute and negatively impact the environment at will. But if some of these framings are wildly imaginative and conspiratorial, or merely cynical and denialist, they nonetheless signal an additional problem in public discourse, which is the high levels of distrust in the sincerity of political rhetoric, about the entanglements of political power and private capital, and in public discourse about climate policy. ^11^ Moreover, the mainstreaming of climate awareness has led—perhaps unintentionally, perhaps not—to “greenwashing” at the corporate level, which has largely voided of content and urgency the message of many ecological demands. In other words, the response (or lack thereof) to climate change is yet another important issue that is often weaponized as part of the oppositional logic of “the people” vs. “the elites.” ^12^ The two novels under discussion, by their portrayal of climate disasters that are not only certain but very near in the future as a result of the “present course of destructiveness” ( Lawrence, 2020: 318), also offer interesting reflections on the potential outcomes of such a lack of action and, perhaps, of imagination.

## Drowning and thriving in the Capitalocene

If in Robinson’s *Mars Trilogy* an extraordinary rise in sea levels becomes “an eco-social event that catalyses change,” forcing humanity to collaborate and eventually resulting in revolutionary social change ( Cooper, 2021: 244), in *New York 2140* the rise in sea level gives way not to revolution but to renewed and lucrative business models in a thriving Capitalocene. The novel is set in a flooded Manhattan in the year 2140, well after the devastating effects of two catastrophic events of sea level rise, called the First and the Second Pulse. These events were followed by periods of famine, economic crisis, and people fleeing from coastal cities to the interior; in the year 2140 Denver, Colorado, is the new capital city of the United States, as Washington D.C. has become uninhabitable. The migratory flows also meant the temporary loss of New York’s status as the capital of global finance, but in the opening of the novel the city is thriving again, even if Lower Manhattan remains permanently flooded and Midtown is an intertidal zone. The city has resurged as a bustling “SuperVenice, majestic, watery, superb” ( Robinson, 2017: 6), according to one of the narrators in Robinson’s multifocal narrative. The resurgence also means that the city is ripe for the next real estate boom. The flooded areas in Lower Manhattan and Midtown—which had been abandoned in the past, were then taken over by scavengers and squatters, and eventually rehabilitated by groups of citizens who established cooperatives and communal forms of living that include farms on the rooftops—are now ripe for gentrification. They are being targeted by the vulture capitalists uptown, whose aggressive takeover bids also include harassing tenants and sabotaging buildings in order to expel them. In short, the city keeps happily marching on to a new cycle of bubble, bust, and bailout; nothing has changed even if everything has changed. The city embodies the triumph of the neoliberal Capitalocene, having adapted with great flexibility to all the challenges brought about by extreme weather and sea-level rise (which it itself has caused): canals of polluted, oily water are navigated in water taxis and *vaporettos*, while skybridges connect the skyscrapers whose ground floors have been turned into boathouses. The cityscape is also teeming with wildlife, with oyster farms in the canals and an abundance of fish, fowl, beavers, muskrats, and seals. In the rest of the country, many species have become extinct or are on the verge of extinction, but a process of rewilding—which doubles as a lucrative TV show—is underway, forcing the migration of buffalos and polar bears to new habitats.

There is an unmistakable irony in this upbeat description, as it is far from some imagined, recovered Arcadia: as the novel makes increasingly clear, the effort to restore the balance of the ecosystem has become wholly integrated with the flows of finance capital and is contingent on the availability of bankable or monetized solutions: as noted, the assisted migration program is also an extremely popular reality show, the de-extinction of mammoths is justified solely by commodification, as there is a need to lower the pressure on the demand for elephants’ ivory tusks; the Intertidal Property Pricing Index, which tracks sea level change and predicts extreme weather events like tsunamis and storm surges, is used by traders and investors to value drowned assets and to buy derivatives on underwater mortgages, which are fuelling the next real-estate boom. In short, the thrust behind New York in 2140 closely resembles Moore’s notion of capitalism as world-ecology, which stresses precisely how financialization has permeated every aspect of human life (in the sense that, for example, pensions, retirement, healthcare, or life insurance are not only financial products in and of themselves but they in turn generate new derivatives that are speculated on in the global financial market). But the novel is also reminiscent of what Neil Smith (2007) has called “nature as accumulation strategy”: the efforts to mitigate the effects of climate change, in the shape of environmental regulations and requirements, give rise to new industries like the selling and purchase of carbon credits, reforestation quotas, negative emissions, catastrophe bonds etc., which generate huge profits that are, in turn, also financialized. Smith’s notion draws attention to how the rise of these industries meant to remediate the effects of the climate crisis in fact keep the financial wheel and the ecological crisis spinning: you can buy reforestation or carbon credits and keep polluting or chopping down trees because it is more profitable to pay someone else in the Global South to plant the required trees for you. As Ortiz suggests, the neoliberal ecology relies on these “strategies of accumulation by dispossession” ( Ortiz, 2020: 267) and the practical demands that arise as a result of climate change actually enhance “the dominance of the financial sector over the overall system, as more opportunities for commodification through financialization are created by the climate crisis itself” ( Ortiz, 2020: 266). It is, as Ortiz calls it, a “deadly symbiosis” ( Ortiz, 2020: 266) between nature and urban-centered finance capital, one for which the novel offers a seemingly impossible, radical and revolutionary resolution.

Two of the novel’s main focalizers, Mutt and Jeff—coders and drifters who are also the namesakes of a long-standing American comic strip—understand better than most that in the New York of 2140 everything is interconnected globally—Manuel Castells’ notion of the network society (1996) is an apt description here—but in such a tight, material and seamless manner that “defectors can’t get outside it” ( Robinson, 2017: 6). Even more so, a type of managerial and technocratic state has successfully blunted any desire for or expectation of political action on the part of the citizenship ( Swyngedouw, 2010). For Mutt and Jeff, it is only by understanding this global financialized and technocratic network, and its absolute totality, that a glitch in the system can be found and resistance can be effected. The urban space of *New York 2140* becomes thus, through the perspective of these two characters, a potentially legible text, a situational representation of global, financial capitalism through which it is possible to read the neoliberal world-ecology and our place within it. Mutt and Jeff, who live on-and-off the grid, spend the entire novel trying to find a glitch that will bring the global financial system down. The perspectives of the novel’s seven narrators or focalizers converge in the final sections, when a devastating hurricane hits the city and, in its aftermath, a horizontal logic of resistance gains impulse: Citizens of the intertidal succeed in coordinating, first locally, then globally, a debtors’ revolt, as people stop paying rent, mortgages, loans, credit card debt, etc., causing the predatory financial sector to crash and banks to be nationalized, in a reverse movement of what happened in 2008 when they were bailed out by the Federal Reserve. By refusing to inject capital into the system, a sort of dual power is established, very much in the way Jameson imagines in his manifesto *An American Utopia* (2016): ^13^ The multinational capitalist dominance eventually withers away, deemed no longer necessary, against the already-existing networks of cooperative ownership of buildings, self-sufficiency and communal living in the flooded areas. ^14^

This type of utopian possibility is qualitatively different from the one Robinson imagines in the short story “Mutt and Jeff Push the Button,” which is Robinson’s contribution to Jameson’s *An American Utopia* and which shares these two characters with the novel. In the short story, Mutt and Jeff “push the button” that will “end capitalism” by hacking into the computers at the World Trade Organization and rewriting “the code,” which is the “set of stupid laws” ( Robinson, 2016b: 100) that rule global financial capital. In rewriting them, they introduce “the opposite of efficiency, which is to say, justice” ( Robinson, 2016b: 104); the rewritten laws include, among other things, Jameson’s idea of universal drafting. If the story’s “solution” is cheekily simple, Robinson’s novel does take up Jameson’s call to think in utopian terms, as well as to re-politicize the issue at hand, which means really thinking about “how we get to a better system” (Robinson in O’Keeffe, 2020), how we get from here to there. Jameson has repeatedly argued that dystopia has become our preferred speculative mode precisely because we live in a world in which “we can no longer imagine the future” ( Jameson, 2016: 13); in contrast, both Jameson and Robinson have always argued for utopian writing and thinking as a way of “saying NO” to the here and now (or what Moylan referred to as the critical utopian gesture of “negating the present”) while also thinking about alternatives to a present which is not necessary but contingent; in this, we can envision the future as disruption.

If in the short story “Mutt and Jeff Push the Button,” the button magically rewrites or edits the fourteen laws of global financial capitalism, immediately changing social organization and economic-political relations, the ending of *New York 2140* contains, however, a warning against such magical or easy solutions. Not incidentally, magical thinking is a feature and not a bug of twenty-first century populist rhetoric, and part of what explains its appeal, offering uncomplicated “analyses” and straightforward “solutions” to what are otherwise complex, multi-layered and often intractable social, political and/or economic problems. Simplistic “solutions” such as Brexit’s “Take Back Control,” Donald Trump’s “Build the Wall,” or former UK Prime Minister Rishi Sunak’s “Stop the Boats,” will not, in essence, solve any of the problems that “the people” are facing. In a similar way, the discourse of eco-efficiency ( Martínez-Alier, 2002; McDonough & Braungart, 2002), with its emphasis on continued economic growth compatible with sustainability, offers the alluring possibility of mitigating the so-called “negative externalities” of such growth through capital-based stopgaps, without addressing the urgency and the level of magnitude of the change that needs to happen to avert disaster. Wealthy corporations amassing profits in the Global North can pay farmers in the Global South to plant trees as carbon offsets; passengers can pay a small fee to offset their carbon emissions and continue to fly cheaply and often needlessly (when more environmentally friendly alternatives do exist). In other words, magical solutions are not only easy but require less work, and they also save us from struggle or disappointment, two of the most effective ways to blunt political and civic agency. And by not targeting the root cause of the problem, they are also ineffective to change the status quo.

Robinson’s novel seems to be acutely aware of this, as well as of the tremendous pull of the Capitalocene, and the ending of the novel reminds us of the constant struggle that will be needed to uphold any gains that might have been achieved. As one of the narrators notes,

there was no guarantee of permanence to anything they did, and the pushback was ferocious as always, because people are crazy and history never ends, and good is accomplished against *the immense black-hole gravity of greed and fear*. Every moment is a wicked struggle of political forces, so even as the intertidal emerges from the surf like Venus, capitalism will be flattening itself like the octopus it biomimics, sliding between the glass walls of law that try to keep it contained…. ( Robinson, 2017, 604; emphasis added).

Despite this final warning, critics like Andrew Milner have argued that *New York 2140* is an eutopia that is “betrayed by its utopianism,” because if there have been “no significant changes to either the American constitution or the banking system,” which has repeatedly been bailed out between 2008 and 2140, “[h]ow realistically likely is it that all this entrenched power could be effectively challenged as a result of one hurricane, no matter how devastating?” ( Milner, 2020: 88). In Milner’s view, this utopian ending, however contingent and however deliberate on Robinson’s part, does indeed “come far too cheaply” ( Milner, 2020: 90), as if pushing a button, not sufficiently plausible (or logical) in intratextual terms.

## Utopia can wait

A somewhat different take is offered in Beckett’s *America City*, although the novel also hinges on the exploitation of “the calculus of dread and comfort” ( Beckett, 2017a: 93), in this case by populist political operators. In his blog entry “Utopia Can Wait” ( 2021) Beckett shares his skepticism about the demands of the “more radical wing of climate change activists”—an end to greenhouse gas emissions and an end to capitalism as its condition of possibility—because “there is absolutely no way that a completely new and fully functional political and economic system is going to be constructed in the next few years” ( Beckett, 2021). Beckett calls for less “radical heroics” and more “meticulous practical work […] on problems such as mass energy storage, affordable green fuels, and carbon neutral cement” ( Beckett, 2021). This accords to Milner’s typology of recent climate fictions as trending increasingly towards adaptation ( Milner, 2020: 80–81) and poses questions regarding the urgency felt in those regions that are in more precarious and vulnerable positions (usually, poorer countries), that do need “radical action” in order to have a chance to survive. But Beckett is perhaps correct in identifying that “the political and business headaches that come with them” ( Beckett, 2021), i.e. with giving course to those demands, may be the biggest challenge still.

In thinking about “where we may be headed,” Beckett constructs a near future that is clearly recognizable, reinforcing the links between near-future SF, climate fiction, and realism. Thus, he seems to concur with Robinson’s diagnosis that all near-future science fiction will probably be climate fiction. Beckett’s cli-fi world building is a clear example of how the “future” has become the present, as there is no “abstracted other place” ( Moylan, 2014: 6) but the here and now: the United States of the next few decades, which has undergone a civil war after a catastrophic climate event and is once again on a populist drift. Interestingly, the question of *how* to write about climate change in a way that is somehow useful is at the forefront of Beckett’s project, as he considers the spectrum between optimism and hope, between eutopia and dystopia, and what may move readers to action: “Appallingly bleak scenarios probably just encourage fatalism, while heartwarming stories of people in the future rebuilding civilisation from scratch after a catastrophe can seem positively appealing” ( Beckett, 2012) and blunt the sense of urgency. Indeed, this is one of the key questions when considering eutopian imaginations; as Mathias Thaler puts it, how can social dreaming be empowering without being escapist wishful thinking? ( Thaler, 2019: 607). But as Thaler suggests, hope and despair are not polar opposites but can work together in complex ways that make them politically relevant, and this is the task of what scholars, including Moylan, have defined as the “critical dystopia.” This narrative has a “dual function,” which is, on the one hand, to “determine where danger looms in the present” and to “gesture towards potential responses in the future” ( Thaler, 2019: 608). For Thaler, they key characteristic of critical dystopias is that they

pivot around a type of hope that remains sensitive to the catastrophic failures of the past and alert to the immense perils of the present, without, however, foreclosing the prospect of a less oppressive, less violent, and less unequal future. ( Thaler, 2019: 608)

It may be argued that, between the poles of hope and despair, Beckett’s narrative seems to be moving closer to the latter—especially because of how recognizable, and sometimes even prescient, his descriptions of technological domination, the manipulations of social media, and its associated “algorithmic outrage” are. While Robinson’s utopian narrative leads to a possible socialist future, Beckett’s dystopian trajectory leads to Trumpism and Brexit. Although Beckett started working on the idea that would give rise to *America City* back in 2012 (a short story titled “Destiny”), when he got back to it in 2016 he did so “against a new backdrop of real political events: first the Brexit vote in the summer of 2016, and then the rise of Donald Trump” ( Beckett, 2017b). Beckett offers some insight into how his creative process unfolded:

Obviously the book is deeply influenced by my thoughts about why and how these things happened and what I felt I was learning about how politics work. I felt very challenged by the fact that the 2016 Presidential election was going on as I wrote the book. Here I was, writing about an American politician winning an election by appealing to the tribal instincts of voters, and meanwhile in real life… In fact, in some respects what was happening in reality was *stranger* than what was happening in my book. I’m biased of course, but if I compare Slaymaker and Trump, it’s Trump who seems more like a made-up character. So there was a while there when I felt like the real world was overtaking me on the inside lane. ( Beckett, 2017b; emphasis in the original)

A familiar feeling many writers have expressed since 2016, and that seems to have moved up a notch in 2024. *America City* is set in the United States roughly a century from now when, as a result of ongoing climate change, storms and hurricanes hit the coast every year, while vast areas of the interior in the south have become desertified and have insufficient water. Farmlands are no longer viable in those areas and their impoverished towns and cities cause a steady stream of climate refugees to head north every year. This internal migration receives an increasingly hostile welcome up north, where these climate refugees are disparagingly called “storm people” or “barreduras,” ^15^ and some northern states begin to consider imposing internal border controls to keep them out. The xenophobia that is typically directed at the foreigner is now fair game within national borders. The southern people themselves know this (even as they reproduce their own national biases): “It’s like we’re not Americans anymore […]. It’s like we’re Mexicans or Haitians or something” ( Beckett, 2017a: 36). As becomes clear, the anti-migration discourse pushed by the politicians in charge is not really about ethnic, racial or even cultural concerns, but more crudely about economics. This, as Zachary Levenson (2017) convincingly argues, is a key manoeuvre of populist obfuscation, where class interests are disguised as genuine political, social and/or cultural anxieties about national identity and national sovereignty. ^16^

In the midst of all of this, a British PR professional living in the United states, Holly Peacock, begins working for a charismatic US Senator, Stephen Slaymaker, a “ferocious American nationalist, [a] bloodstained warrior from America’s wars in the African Copper Belt, [a] self-made man who thought that just because he’d been able to claw his way up from nothing, then everyone else could too” ( Beckett, 2017a: 15). He is the proverbial outsider who makes it into politics—the same appeal, however false, claimed by figures like Trump and Milei—, a man who built the biggest trucking company in the country out of humble origins by his effort alone, the embodiment of that irresistible American ethos of social mobility through sheer individual effort. He also has a track record of denying anthropogenic climate change (which is simply the result of “God’s will” [ Beckett, 2017a: 28], very much in line with the most fundamentalist wing of the Christian right). Slaymaker decides to run for President under an ambitious program (“Reconfigure America”) that is meant to move the American population northward and thus remedy the north-south divide that is, once again, tearing the country apart: The internal migration of the dispossessed clashes against the interests of those who will soon be precarious too, and in the background there is an allegedly progressive, professional and educated class who is so detached from these material realities that they are disparagingly called “delicados.” Empathy is dangerous when survival is at stake; as one of the narrators admits, “It’s dangerous to feel sorry for [the barreduras], for that might mean an obligation to help them” ( Beckett, 2017a: 92). It may also require acknowledging and addressing the root causes that drive them north. Slaymaker’s relocation program is not motivated by any sort of humanitarian empathy either, but by pure political opportunism ^17^ and economic dogmatism: The people in the interior and on the Storm Coast are “soaking up tax money,” living where they can’t be productive and are thus “a drain on the country’s resources” ( Beckett, 2017a: 23). These opinions are echoed in the online public sphere, which Holly checks incessantly and is very adept at manipulating: “Fuck the storm people. *Fuck* them. If they’re dumb enough to still live in those places, how is that our problem?” ( Beckett, 2017a: 33; emphasis in the original).

Holly’s job is to “sell” the migration program to the people, especially northern voters, who have to be convinced that welcoming millions of climate refugees from the south is in their best interest. She finds a way that involves digging into the oppositional logic of “us versus them,” but displacing it towards the exterior: The neighbor up north, Canada. The affective logics of populism that both Holly and Slaymaker understand so well are put to play “to get America angry” ( Beckett, 2017a: 188). The plan is to rile people up against Canada so as to justify a war: “a bit of hostility toward Canada may not be a bad thing. Americans need somewhere to direct their frustrations other than to one another” ( Beckett, 2017a: 189). Building on a sense of grievance, on Canada’s “greediness” and “lack of solidarity,” Slaymaker’s team organizes a mob (of northerners and “barreduras”) and buses them to the Alaskan border under the false promise that Canadian authorities will let them in. Eventually the mob overrides the Canadian border forces, shots are fired, and chaos ensues. The stage is set, and Slaymaker sends tanks over the Alaska border and “bomb[s] the Ministry of Defense in Ottawa, reducing it to rubble” ( Beckett, 2017a: 333). The war eventually comes to an end with Canada’s defeat and utter destruction ( Beckett, 2017a: 334) and the annexation of a territory called “Northland,” the capital city of which will be America City. Holly knows the symbolic importance of this development, which allows for a reframing of the classical frontier mentality by which the new “pioneers” will settle across the border—people who had been until very recently “foreigners in their own country” ( Beckett, 2017a: 328), “people who’d felt humiliated and powerless, and finally they were being given some hope and something to be proud of” ( Beckett, 2017a: 199). As with all nostalgic retrotopias, the movement is built on the hopeful resurgence of the myths of the past—references to Lincoln, the Civil War, and the western frontier abound—, as that which gives meaning to and articulates a new sense of collective pride and worth, which is one of the key promises and allures of far-right twenty-first century populism (MAGA, MEGA, and all its variations).

By the end of the novel, Holly becomes disillusioned with how it has all turned out, as she can no longer justify Slaymaker’s populist agenda as something meant for the greater good. She is also fully aware that if nothing is done, climate change will continue to render land uninhabitable and pushing populations further north, until “there’s nowhere left to go and your precious America will finally be completely fucked, along with the rest of the world” ( Beckett, 2017a: 337). But, as the narrator recognizes, there is no political will to be summoned, no will to implement the necessary measures to avoid catastrophe. Shockingly, but perhaps not surprisingly, the novel ends with Slaymaker’s political assassination at the hands of a young Inuit, a new “foreigner” in his own land (that has now become Northland). ^18^ The reference is succinct, just four paragraphs, but the spectral presence of the Inuit at the end of the novel (and also in the brief Afterword) is perhaps the one act of political agency and resistance in a novel that details how the populace can be manipulated at will by populist rhetoric, however doomed the gesture might be. Alternatively, we can read this final engagement with the indigenous as pointing in the direction that many scholars ( Whyte, 2018; Colebrook, 2019b; Heise, 2024, for instance) have observed in their critiques of most recent climate fictions: That the inability to imagine “the end of the world” (i.e. of the capitalist, imperialist, and extractivist part of it) gives rise to the occasion to mourn the loss of “other” worlds, *a* world that is not *the* world ( Colebrook, 2019a: 44), thus foreclosing the possibility of imagining alternative, radically different futures that are not necessarily tied to the survival of the Western paradigm. “ *This world that is the world* is the world of a narrating and global ‘we’ who can look upon the loss of other’s worlds. Extinction, genocide, erasure of a way of life: when these occur elsewhere they are occasions for lament” ( Colebrook, 2019a: 44; emphasis in the original). In Beckett’s novel, the Inuit have been overrun in every sense of the term—annexed, moved into, and they are soon to become exotic tokens as the whole of the United States moves into the Arctic: the reference to Holly owning “a tiny nativity carved in Inuit style from a piece of soapstone” ( Beckett, 2017a: 357) is not gratuitous. It is precisely the “end of the world” that might offer indigenous peoples “a chance for existence” ( Colebrook, 2019b, 277), but the novel seems to suggest otherwise, and in this I read a sort of pessimistic realism that is consistent with the “fidelity to the real” that *America City* displays throughout. If the revolutionary act at the end of Robinson’s *New York 2140* comes out as a bit implausible, there is nothing implausible in *America City*’s imagining the United States taking over Canada, Northland, or Greenland for that matter, by force, and causing the extinction of yet more peoples, cultures, species and environments.

## Some concluding thoughts

Climate change *is* the end of the world but, as Colebrook suggests, in the twenty-first century of Trumpian politics even “the end of the world can be trumped. Not only are we entering the sixth mass extinction, but we are doing so in a way that intensifies the barbarism that got us here” ( Colebrook, 2019a: 45). The acceleration of a technocratic and financialized Capitalocene into the cruelty and contradictions of the Trumpocene implies not only the framing of climate action and awareness as a globalist hoax and as an indulgence of the well-off at the expense of the precaritized working classes, but also the sequestering of public and civic attention even when the end of the world does become *the* number one emergency. The aggressive, erratic and, sometimes, merely performative actions of elected and non-elected leaders like Trump, Milei, and Musk (which Steve Bannon eloquently explained as “flooding the zone with shit”) distract us from keeping tabs with the real and powerful global players who are fueling (and funding) the march towards impending environmental disaster, as Colebrook suggests, and potentially blunts our response. Additionally, in a world of utter relativism, fake news and alternative facts, we run the risk of becoming blind to both the past and the future of extinction, of the history of capitalist and imperialist exploitation that has created and intensified the conditions—environmental, economic, political, social—that have got us here in the first place ( Colebrook, 2019a, 46). Occlusion, erasure, blindness, confusion: these are the markers and tactics of the Trumpocene. An even cursory look at recent events shows this.

In late July of 2024, a British-born young man went on a killing rampage at the seaside town of Southport, west of Liverpool in England. He stabbed and killed three young girls and injured a dozen others, including their teachers. Even before the motives and identity of the perpetrator where confirmed by the local police, far-right agitators were quick to capitalize on the heinous crime and launched a powerful disinformation campaign that claimed that the killer was a recent refugee and stirred a mob to two weeks of riots which extended to other cities. Rioters tried to torch a hotel sheltering asylum seekers in Rotherham, while in another town “they ransacked a police station and burnt out a Citizens Advice Bureau. In Belfast, they destroyed local shops, including one set up by a Syrian refugee and another by an immigrant from the Middle East, and broke into homes. In Liverpool, Hull, and Plymouth, they injured police officers. In Leicester, they menaced neighbourhoods, with Muslim residents fearful of leaving their houses” ( Lucas, 2024). As an editorial for *The Guardian* put it at the time, the mobilization and weaponization of mob violence by the British far-right relied on recognizable themes: “a distrust of those in power, a willingness to blame economic and social ills on outsiders, on migrants and, in particular, Muslims, and the willingness of the cynical and the shameless to use these situations for their own profit” ( The Editors, 2024). As has been sufficiently argued, the British Conservative party has significantly contributed in recent years—especially since the Brexit vote—to make the fringe mainstream with its inflammatory rhetoric and culture war agenda and has left Britain ripe for this type of xenophobic, chauvinistic and populist reaction.

An eerily similar incident had taken place in the Republic of Ireland in November 2023, when three children and a woman were stabbed outside a primary school in Dublin city center; while the Garda (local police) kept the perpetrator’s identity undisclosed, ^19^ misinformation quickly spread through Whatsapp, Signal, and Telegram about the identity of the attacker as an “illegal immigrant” and about the children being dead. Around 6pm, mob violence by far-right and anti-immigration agitators escalated, which led to a night of riots that included torched buses, looted shops, over sixty Gardaí assaulted, and certain neighborhoods being cordoned off. The level of violence was unprecedented in Dublin’s modern era, according to the Garda. In Spain, just two weeks after the events in Britain, the deadly stabbing of an 11-year-old while he was playing football at a municipal pitch became the excuse for similar campaigns of misinformation and criminalization of foreigners, especially those who resided in a nearby center for migrant children. That the perpetrator was a Spanish national was irrelevant to those intent on spewing misinformation and anti-immigration rhetoric and to right-wing political parties that repeated it. When the family of the victim asked the public and the media not to make such an unwarranted association between the attack and those seeking asylum, they were mercilessly abused on social media by the very same people that, in theory, were only concerned about their safety.

Events like these keep repeating themselves across the West, where the far-right fuels and capitalizes on anti-immigration sentiment, linking migration figures to a decrease in living standards and dwindling opportunities for ever larger sectors of their populations and stoking fears of cultural replacement. It is not uncommon for commentators to explain away this type of anti-immigration violence and rhetoric as the product of legitimate socioeconomic concerns or cultural fears, falling in the trap of far-right agitators and xenophobes who exploit and weaponize such vulnerabilities. The immediate association between socioeconomic precarity, lack of opportunity, and far-right affiliations as transparent and inevitable can and must be refuted, especially because it selectively ignores the millions who do live in precarious socioeconomic conditions and do not turn into thugs or violent xenophobes. The pertinent question, at least for the Left, should be how to counter the encroachment of precariousness and extreme inequality into ever-larger swaths of the population, in what are otherwise affluent societies, questions that are at the center of notions like “precarity capitalism,” “neofeudalism,” and “technofeudalism,” as previously noted. Moreover, as Apostolidis demonstrates, relentless socioeconomic precarization can pose real and material impediments to political literacy and agency, as it tends to block people “from developing the critical dispositions” and the time needed for exercising “democratic citizenship” ( Apostolidis, 2022: 114). A precaritized existence can in turn facilitate the appeal of xenophobic rhetoric even across lines of class, gender, and ethnicity, which explains why racialized, migrant and/or gendered people can sign on to the white supremacist, ethno-nationalist and isolationist discourse of Trumpism, with no apparent contradiction ( Apostolidis, 2022: 116). On the other hand, whether we consider today’s migratory flows the natural result of a process of market liberalization and neoliberal globalization since the 1990s, exacerbated in recent decades by increased inequality and wars across the globe, it is hard not to argue that the next great migratory movements will be climate-induced, the result of extreme weather events and continued climate change, if it they are not happening already, as many reports by international organizations suggest ( Center for Global Development, 2022; International Organization for Migration, 2021; UN, 2018; World Bank, 2021). In focusing on two novels where climate change is the main driver of migratory (and financial) flows, I have sought to illustrate how climate fiction can also bring into sharp relief—in a sort of mirror image—how quickly a given set of conditions can accelerate; if climate emergency is understood as the ultimate precarity, we can more easily read the rapid transition from deteriorating socioeconomic conditions to a devalued political culture to xenophobia, nativism and, maybe, all-out-war.

In the expectation of all these certainties—the acceleration of financial, technocratic capitalism in the age of AI, ongoing precarization in a world dominated by the uber-rich, the rise of authoritarianism and isolationism, the increased exposure to climate change and resulting forced migration—the next relevant question is how to develop a type of politics that can actually contest the rising wave of Trumpism and other exclusionary forms of xenophobic populism, which will not actually provide solutions to these challenges. If these questions are best left to sociologists and political scientists, and they have in turn shown the usefulness of reading fiction as theory, my aim in this article was not, by any means, to provide such answers but to illustrate, through a joint reading of Robinson’s and Beckett’s novels, how literary fiction can contribute to making legible this contested present. It is the role of fiction not only to imagine alternatives and futures, but to add nuance and depth to realities that might be difficult to capture in their full complexity, especially when obfuscation, misinformation, and easy solutions are the order of the day. From the close proximity of the near-future, *New York 2140* and *America City* manage to capture a present that is both Capitalocene and Trumpocene—or, as I have suggested earlier, the latter as an iteration of the former—and, through their different utopian impulses, offer vivid speculations about the future. Robinson’s *New York 2140* insists on the importance of utopian thinking to keep open the possibility of a transition to post-capitalism; while there is no radical action offered in terms of the climate, just adaptation, we can imagine that the “withering away” of the capitalist order would entail some sort of reversal in this sense. Beckett’s *America City* offers a darker rendering through a narrative that is so close to the present that, had it not been written before 2016, would make us question its labelling as science fiction. I have also argued that their being both climate fictions serves to underscore not just the authors’ concerns about the effects of climate change in the near future but to illustrate, via the exposure to extreme weather events and forced migration, some of the expected outcomes of the Capitalocene and the Trumpocene: an increasingly unequal society where the majority is made precarious, under the continued exploitation of both people and nature and endless commodification (Robinson), or the regression into a nativist, fractured and isolationist national entity that can only envision imperialist expansion and aggression as the way out of relentless devastation. Crucially, their utopian and dystopian inflections also mark differences as to the level of agency that humans can exercise: While in Robinson’s utopia the thrust of collective action somewhat implausibly remains alive, amid the would-be blunting effects of the Capitalocene totality, in Beckett’s dystopian trajectory only political violence seems to remain, when the collective will has been so thoroughly manipulated and perverted. Each in their own way, these two novels speak loudly to the present, inviting us to tread the line between utopia and dystopia, between magical thinking and revolutionary alternatives, and to consider, above all, how rhetoric and storytelling can be used both for laudable or nefarious ends.

## Ethics and consent

Ethical approval and consent were not required

## Data Availability

No data are associated with this article.

## Footnotes

^1^ To name but a few well-established examples: Margaret Canovan, “Trust the People! Populism and the Two Faces of Democracy” ( *Political Studies*; 1999, 47: 2–16); Margaret Canovan, *The People* (Polity Press, 2005); Ernesto Laclau, *On Populist Reason* (Verso, 2005); Cas Mudde, “The Populist Zeitgeist” ( *Government and Opposition*; 2004; 39.4: 541–563); Slavoj Žižek, “Against the Populist Temptation,” ( *Critical Inquiry*; 2006, 32: 551–74).

^2^ Besides the already-mentioned volume by Albertazzi and McDonnell (Palgrave Macmillan, 2008), see the more recent Cas Mudde & Cristóbal Rovira Kaltwasser, *Populism: A Very Short Introduction* (Oxford UP, 2017); Chantal Mouffe, *For a Left Populism* (Verso, 2018); Heike Paul, Ursula Prutsch & Jürgen Gebhardt (eds), *The Comeback of Populism. Transatlantic Perspectives* (Universitätsverlag Winter Heidelberg, 2019); Duncan McDonnell & Annika Werner, *International Populism. The Radical Right in the European Parliament* (Hurst, 2019); Aurelien Mondon & Aaron Winter, *Reactionary Democracy. How Racism and the Populist Far Right Became Mainstream* (Verso, 2020); Steven Forti, *Extrema derecha 2.0. Qué es y cómo combatirla* (Siglo XXI, 2021).

^3^ This is a classic appeal to “the people” adapted to the local idiosyncrasy. A literal translation from Spanish would be misleading and offer no clarification (“Argentines who do good”? “Argentines of good breeding”? “Honest Argentines”?); the appeal is both willfully blurry and class specific. The lack of definition of the group (just as in the case of “the people”) helps to keep the appeal open for various people to identify with and feel themselves being interpellated. My interpretation of this phrase (in the context of it being used by Milei and considering his politics) is “Argentines who are orderly, hard-working, and deserving—i.e. not poor—, because they contribute to rather than being a drain on the state.”

^4^ See, for example, Barry R. Posen, “The Rise of Illiberal Hegemony” ( *Foreign Affairs*, 13 February 2018); José Luis Valdés-Ugalde, “Hegemonía no liberal” ( *Norteamérica*; 2019, 14.2: 101–120); Donald E. Pease, “Donald Trump’s Settler-Colonist State (Fantasy): A New Era of Illiberal Hegemony?” (in *Trump’s America*, ed. Liam Kennedy [Edinburgh UP, 2020]: 23–52); Michael J. Boyle, “America and the Illiberal Order After Trump” ( *Survival*; 2020, 62.6: 51–76); Donna M. Goldstein, Kristen Drybread (eds.), *Corruption and Illiberal Politics in the Trump Era* (Routledge, 2023); Steven Hahn, “The Deep, Tangled Roots of American Illiberalism” ( *The New York Times*, 4 May 2024).

^5^ The Gadsen flag originated in the American Revolutionary War of 1775 and bears the motto “Don’t Tread on Me,” which helps to explain its repurposing as a libertarian symbol in the United States: the denunciation by American colonists of their oppression by the imperial master (Great Britain) in the eighteenth century is turned into a libertarian rallying cry against the US federal government in the twentieth. Its popularity among certain right-wing populist groups outside the United States in recent years signals a certain level of mutual recognition and ideological coordination among voters and supporters of the new reactionary right, who distrust “the State” and all established institutions.

^6^ For example, novels like George Saunders’s *Lincoln in the Bardo* (2017) and Viet Thanh Nguyen’s *The Sympathizer* (2015) can be read as clear-eyed reflections on moments of paradigm shift even if not explicitly engaging with the United States of Trump.

^7^ Elon Musk’s vision for the “Department of Government Efficiency” (DOGE) immediately comes to mind.

^8^ See also Jameson, 2016: 3.

^9^ “And America was a pirate ship too, of course, but at least for a moment it was pretty much afloat” ( Beckett, 2017a: 7).

^10^ Schmidt challenges this view and demonstrates through discourse analysis that the new climate movement (as exemplified by the activism of Greta Thunberg) makes use of both a populist framework (here understood solely as rhetorical style, not as ideology) and a depoliticizing strategy (the post-political here understood as universalizing claims that assume the absence of conflict and dissent). Their argument draws largely from Erik Swyngedouw’s provocative notion of “post-political populism” in relation to the depoliticization of climate change activism. See Erik Swyngedouw, “Apocalypse Forever? Post-political Populism and the Spectre of Climate Change” ( *Theory, Culture & Society*, 2010; 27[2–3]: 213–232).

^11^ Indeed, this is one of the five characteristics that Gregers Andersen identifies in cli-fi: the motif of “the conspiracy”: “anthropogenic global warming does not just appear as an object for objective (apolitical) science to investigate, but also as a narrative construction and political instrument [...] this is an epistemological issue that again and again emerges in the climate fictions [...]. In these fictions conspiracy is exactly what brings the entangled and complicated relationship between science and politics, truth and ideology to light.” (Andersen G: *Climate Fiction and Cultural Analysis: A New Perspective on Life in the Anthropocene*. Routledge, 2019: 64).

^12^ Or, as Greta Thunberg would have it, “our generation” (i.e. the young) vs. the “world leaders” (i.e. politicians, the older generations, irresponsible adults).

^13^ *An American Utopia. Dual Power and the Universal Army*, edited by Slavoj Žižek (Verso, 2016), is a provocative manifesto by Fredric Jameson followed by ten (mostly dissenting) responses by renowned Marxist scholars, such as Jodi Dean, Agon Hamza, Alberto Toscano, Žižek, and Robinson himself. Jameson’s text can be best understood as a thought-experiment meant to address our inability to imagine an alternative to (neoliberal) capitalism; it proposes a military model of collectivity based on universal conscription to the army that, in exchange for four hours of work a day, would fill in those social spaces that the neoliberal State has neglected or abandoned (for example, free clothing, food, healthcare, and housing, full employment, guaranteed basic income, enough paid leisure time, all of them services that current armies already provide). This universal army would function as a dual power, that is to say, a parallel power structure that does not challenge the State but that, eventually, would cause the State to become redundant and cease to exist. This proposed utopian model of mandatory and universal conscription, while it may seem too coercive, is meant to work as a sort of “cognitive mapping,” reminding us of the many coercions we already endure as part of our current conditions of existence in a globalized neoliberal economy that is increasingly dependent on precarization. If anything, the benefit of Jameson’s model is that, rather than being concealed and thus perceived as natural—even inevitable—, as neoliberal conditions often are, the coercion intrinsic to the universal army would be visible and transparent.

^14^ I want to credit here an observation made by my colleague Andrea Ruthven at the University of the Balearic Islands and which I haven’t had the opportunity to develop: the importance of water as that which enables connections, relation-ability, and the possibility of forming and sustaining new communities.

^15^ From Spanish, “barredura” means the residue collected in the act of sweeping.

^16^ Likewise, there is no contradiction in the leader of “the people” being a millionaire (such as Trump and Berlusconi), because the designated antagonists are not the wealthy classes but “the political establishment,” to which they claim not to belong.

^17^ “We should be looking at this weather problem as an opportunity,” says Slaymaker ( Beckett, 2017a: 27).

^18^ Not surprisingly because of the United States’s long history of political assassinations (all the way from Lincoln to the Kennedys, Martin Luther King, and Malcom X), of which the recent attempt on Donald Trump’s life at a campaign rally is the latest example.

^19^ Eventually (much later) it was disclosed that the perpetrator was a naturalized Irish citizen of Algerian origin, who had been living in Ireland for over twenty years. However, the hashtag used by far-right agitators was #Irelandisfull, which had emerged in direct reference to recent refugee arrivals (especially after Russia’s invasion of Ukraine in 2022) and a housing shortage that has become especially acute in the last five years, together with an unprecedented cost-of-living crisis.
