# Supplementary material for: Transnational readings in the Trumpocene: Kim Stanley Robinson’s New York 2140 and Chris Beckett’s America City
Source: Open Res Eur. 2025 Mar 24;4:214. Originally published 2024 Oct 9. [Version 2] doi: 10.12688/openreseurope.17107.2 (PMC11969134; doi:10.12688/openreseurope.17107.2)
Supplement: Peer review history

# Reviewer report: Reviewer response for version 2

**DOI:** 10.21956/openreseurope.20656.r52356

**Published:** 2025-04-16

**Authors:** Carl Death

**Affiliations:** 1 The University of Manchester, Manchester, England, UK

Thanks for this revised version and the response to the reviewers. I enjoyed reading it and will recommend to future students and researchers in this area.

If any, are all the source data and materials underlying the results available?

No source data required

Is the work original in terms of material and argument?

Yes

Is the work clearly and cogently presented?

Partly

Is the argument persuasive and supported by evidence?

Partly

Does the research article contribute to the cultural, historical, social understanding of the field?

Yes

Does it sufficiently engage with relevant methodologies and secondary literature on the topic?

Partly

Reviewer Expertise:

International Politics, Climate Change, Climate Fiction, Critical Theory

I confirm that I have read this submission and believe that I have an appropriate level of expertise to confirm that it is of an acceptable scientific standard.

# Reviewer report: Reviewer response for version 2

**DOI:** 10.21956/openreseurope.20656.r52423

**Published:** 2025-04-03

**Authors:** Matteo Nicolini

**Affiliations:** 1 Verona University, Verona, Italy

The article is well written. It is coherent, and the way arguments are deployed exhibits consistency and clarity. Within the area of sci-fi, the choice of both novels and the way they are contrasted, compared, and assessed is decisively original. The author perfectly masters them. The text also engages with relevant methodologies and secondary literature on the topic. To this extent, the author use the text as the main fil rouge so as to introduce several interdisciplinary issues, which evidently intertwine in both novels. I particularly enjoyed the research article, inasmuch it contributes to the cultural, historical, social understanding of the field.

 This is particularly evident when the author engages in a conversation with the narratives perused to stir local communities in the not-so-far future ravaged by climate. Her careful examination of how these are framed by the various actors in both novels reminds me of the concept of  interpretive community. Coined by Stanley Fish, it might provide a rhetorical framework for examining how Peacock and Slay maker deploy their arguments. But the same holds valid for the Southport killing, when the 'far-right agitators were quick to capitalize on the heinous crime and launched a powerful disinformation camping'. The Trumpocene provides a further example of how to deny the anthropogenic effects of global warming in the public sphere. (see Stanley Fish, *Is There a Text in This Class? The Authority of Interpretive Communities*. Harvard UP 1980; *Winning Arguments. What Works and Doesn't Work in Politics, the Bedroom, the Courtroom, and the Classroom*, Harper Collins 2017). If I understand Fish correctly, the group is bound together by a set of “interpretive strategies” (Fish, 1980, p. 14), through which its members assign their preferred meaning to, say, texts, the world, and – for what concerns us here – climate change and heinous events. Evidently, the assigned meaning refers not to its ontology, but to its representation; not to its essence, but to its existence. As there is no settled interpretation in a time of climate change, what really matters are these interpretive strategies. And the author perfectly explains us how this occurs in both novels. The *correct* interpretation thus depends on the “winning arguments” deployed to forge climate narratives (Fish, 2017).

 The further feature of the article points to the triumph of finance and its narrative even after climate change has eventually hit the U.S. Despite our existential crisis, the winning arguments are those of the corporate tricksters (to use the terminology perused by Rogers *Law, Fiction and Activism in a Time of Climate Change*. Routledge 2020), since they promote “elusive” narratives as regards climate change: “we can rely on market forces and technofixes to solve the climate change problem” (Rogers, 2020, pp. 12, 25).

 Finance (and the law that supports it) wins because it convinces that, for example, nature restoration must be tackled preserving our economically constructed paradigms.

 With this, the articles open ups new avenues, such as the tthe “strategic” use of the law by pseudo-scientific narratives. Far from offering innovative responses to the environmental crisis, the law is used to adapting *new* ecological issues to the *traditional* framework. Presuming that a *business as usual* approach might adequately counter the ecological decline, probing the law strategically normalises the ecological catastrophe and embeds corporate tricksters’ interests into a narrative offering no real alternatives to acquisitiveness and exploitation.

 Last but not least, neoliberal ecology reminds me of *ecopiety*, i.e., a “contemporary practice of environmental (or ‘green’) virtue through daily, voluntary works of duty and obligation”. In going green, we perpetuate “the logics of global capitalism and market ideology” (McFarland Taylor, *Ecopiety. Green Media and the Dilemma of Environmental Virtue*. New York University Press. 2019, pp. 3, 5).

 These are only reflexions originated by my reading this piece – as well as suggestions for the author in case she might want to pursue her research further in this field.

If any, are all the source data and materials underlying the results available?

Yes

Is the work original in terms of material and argument?

Yes

Is the work clearly and cogently presented?

Yes

Is the argument persuasive and supported by evidence?

Yes

Does the research article contribute to the cultural, historical, social understanding of the field?

Yes

Does it sufficiently engage with relevant methodologies and secondary literature on the topic?

Yes

Reviewer Expertise:

Law and humanities, Legal geography, critical legal studies.

I confirm that I have read this submission and believe that I have an appropriate level of expertise to confirm that it is of an acceptable scientific standard.

# Reviewer report: Reviewer response for version 2

**DOI:** 10.21956/openreseurope.20656.r52355

**Published:** 2025-03-27

**Authors:** Luke Cooper

**Affiliations:** 1 The London School of Economics and Political Science, London, England, UK

A great read - congratulations!

If any, are all the source data and materials underlying the results available?

No source data required

Is the work original in terms of material and argument?

Partly

Is the work clearly and cogently presented?

Yes

Is the argument persuasive and supported by evidence?

Partly

Does the research article contribute to the cultural, historical, social understanding of the field?

Yes

Does it sufficiently engage with relevant methodologies and secondary literature on the topic?

Yes

Reviewer Expertise:

International relations, political science, nationalism, authoritarianism, global political economy

I confirm that I have read this submission and believe that I have an appropriate level of expertise to confirm that it is of an acceptable scientific standard.

# Reviewer report: Reviewer response for version 1

**DOI:** 10.21956/openreseurope.18488.r45043

**Published:** 2024-10-25

**Authors:** Luke Cooper

**Affiliations:** 1 The London School of Economics and Political Science, London, England, UK

This is a very well written and presented piece that discusses two novels, New York 2140 and America City, and how the images and narratives they present are revealing of contemporary discussions of far right populism and its role as an apocalyptic accelerator in the environmental crisis.  

 I very much enjoyed reading the piece and would like to see it indexed. My suggestions for its development are intended to assist the author in refining the argument. 

 For disclosure, and in the spirit of non-anonymous peer review, I should say that while I have read New York 2140 and a number of other KSR novels, I haven't read America City. I believe I was invited to review this piece because of my Cambridge Review of International Affairs piece, "Worlds beyond capitalism: images of uneven and combined development in Kim Stanley Robinson’s Mars trilogy", though my work on authoritarianism speaks to the Trumpocene analysis. I also note for disclosure that like the other reviewer, Carl Death, I am working in the discipline of International Relations, though, I hope, in a curious and interdisciplinary way, and this may impact the kinds of expectations we have regarding the paper's argument. I am also aware having read his comments before writing my own, that my queries overlap with his. 

 I have the following questions/suggestions for Resano: 

 1. It wasn't clear to me what the argument that the article was seeking to make was. The article feel currently like a very interesting and well written commentary on a series of issues pertaining to populism, apocalypse and their narrative representation in fiction. Relatedly, I didn't get a clear sense of why these particular novels were chosen. 

 2. The choice of New York 2140 to present an argument about far right populism seemed a little question begging to me. Does 2140 really present an image of the Trumpocene? As Resano notes it is, if anything, a technocratic and managerial vision of political order, not something that one would associate with populism. While the discussion of 'simple' solutions is interesting it felt a little at odds with some of the earlier argumentation given that this effort was designed to 'hack' the system in an empancipatory way, which was evidently not a Trumpocene conception. Are there passages where the histories of far right populism are discussed as a factor in the crisis of 2140? If so perhaps these could be discussed to strengthen the argument. 

 3. Is one solution to (2) to be clearer that you are treating New York 2140 as a fictional imaginary of the 'capitalocene' of the future? From your description it seems that America City is by contrast a depiction of the Trumpocene - so maybe there are relatively straight forward edits that can be made to clarify these seemingly quite different images and warnings that the novels present?

 4. The introduction is quite long and quite focused on the conjuncture of 2016 which will be familiar to many readers. I would suggest shortening it and using the space to expand the discussion of the capitalocene and trumposcene which is I think the domain where the article is making its contribution.

If any, are all the source data and materials underlying the results available?

No source data required

Is the work original in terms of material and argument?

Partly

Is the work clearly and cogently presented?

Yes

Is the argument persuasive and supported by evidence?

Partly

Does the research article contribute to the cultural, historical, social understanding of the field?

Yes

Does it sufficiently engage with relevant methodologies and secondary literature on the topic?

Yes

Reviewer Expertise:

International relations, political science, nationalism, authoritarianism, global political economy

I confirm that I have read this submission and believe that I have an appropriate level of expertise to confirm that it is of an acceptable scientific standard, however I have significant reservations, as outlined above.

# Response

**Published:** 2025-03-08

**Authors:** Dolores Resano

**Affiliations:** English Studies, Universidad Complutense de Madrid, Spain

(I am responding to both reviewers together since their reports complemented each other so well). I want to sincerely thank Prof. Carl Death and Prof. Luke Cooper for their extremely generous feedback and for taking the time to review my work in such a conscientious and enriching manner. Their expertise and careful reading has helped me to sharpen the argument of the article and to consider aspects, questions, and sources that I had missed. I am truly grateful for their having engaged with my work in such a collegial manner, especially considering how busy their academic schedules must be. I feel privileged for having had this opportunity to have a “conversation” with experts like them, from whom I learnt so much. Thank you. The following is a (non-exhaustive) list of the main changes to this new version:

- The abstract has been modified to make the aim of the article clearer, and to maintain coherence with the plain language summary.
- The introductory section on populism has been significantly shortened to what is relevant as an introduction to the argument.
- The main argument of the article has been disambiguated and refocused to become clearer in the opening sections, which is to examine how these climate fictions also serve to make legible the present political moment and to illustrate potential, future trajectories.
- The discussion of the Trumpocene and considerations about post-apocalyptic narratives have been expanded.
- All the references that were generously offered by both reviewers have been considered and, whenever possible, included in the discussion.
- The choice of the corpus has been justified (mainly on page 4), especially acknowledging and taking into consideration the arguments made by Colebrook 2019, Death 2022, and Whyte 2018.
- The conclusion has been rewritten to be less vague and anecdotal and to re-cap the main claims made in the reading of both novels, as well as to deliver some tentative answers to the questions posed at the outset of the article.

# Reviewer report: Reviewer response for version 1

**DOI:** 10.21956/openreseurope.18488.r45044

**Published:** 2024-10-23

**Authors:** Carl Death

**Affiliations:** 1 The University of Manchester, Manchester, England, UK

This is an interesting, well-written and thoughtful article on climate change and political populism, drawing out insights from two climate fiction novels set in North America in order to offer reflections on various issues including utopian/dystopian narratives, Brexit and Trump, precarity and populism, the desire for ‘easy solutions’ to complex problems, and the role of stories in contesting regressive political narratives. The article is good at combining theoretical discussion, close analysis of the two novels and broader political reflections, and illustrates and frames the discussion with several vignettes from current affairs.

 I enjoyed reading the article, especially given my own research on climate fiction as a fertile resource for theorizing about climate change, and for diversifying perspectives on race, gender, sexuality, and ecology in world politics.

 The article opens with an overview of populist trends over the last few decades, before introducing the two central novels – America City by Beckett and New York 2140 by Robinson – as examples of fiction that are “tuned to the disturbances of a rising populist wave.” The next section introduces the concepts of cli-fi, the Capitalocene and the Trumpocene, categorizing both novels unproblematically as climate fiction. Whilst noting that Beckett’s novel is more dystopian whilst Robinson’s is more utopian, both are set within worlds that could be described using the concepts of the Capitalocene and the Trumpocene. Subsequent sections discuss the two novels sequentially, giving an insightful summary of the novels which are accessible even to readers who may not be familiar with the originals. Personally, I should note that I have read Robinson’s novel but not Beckett’s. Some concluding thoughts are offered at the end, beginning with more examples of far-right populist mobilizations, and asking what sorts of politics might “actually contest Trumpism and exclusionary types of xenophobic populism?”

 In this review I will suggest two ways in which the paper could be strengthened or clarified – the specifics of the central argument and the differences between the two novels – and three areas that I think are important but neglected.

 The first and most important issue is that I am still somewhat unclear about the core argument of the paper, even after several close readings. In part this seems to arise from slightly different accounts of the argument at key points, but there are also issues with the structure and style of the paper.

 To give some examples of the former: in the abstract it is stated that these novels offer a similar diagnosis of climate pessimism, whereas the plain language summary focuses on Brexit and Trump as important motivations for the novels. The final paragraphs of the introduction focus on the heightened sense of precariousness in both novels and the possible connections this has to populism, whereas the latter stages of the article raise questions about “environmentalism of the poor” and the politics of responding to and contesting Trumpism. I am sure it is possible to connect these related themes into a coherent central argument, but I wasn’t ever really clear what the core claims in this paper were. This is reinforced by the final lines of the paper which remain somewhat on-the-fence: e.g. “Both Beckett’s and Robinson’s novels invite us to tread the line between utopia and dystopia, between magical thinking and revolutionary alternatives, and to consider, above all, how rhetoric and storytelling can be used both for laudable and nefarious ends.”

 The structure of the paper also works against a clear and convincing central argument. The introduction mainly consists of lengthy discussions about populism but states several times that this article is not a contribution to definitional debates. When the novels themselves are eventually introduced we are told that they can speak to debates about not just populism but also precarity, ‘the political’ and climate change, but a clear statement of what the paper will argue is missing. Subsequent sections introduce new concepts and literature and make interesting observations, but even the conclusion is anecdotal in tone and doesn’t manage to convincingly re-state a clear central line of argument.

 The second important area for strengthening concerns the analysis of the two novels. From the description given here it seems that whilst both novels draw on similar themes, the political message and conclusions to the stories are very different, along dystopian/utopian lines. There might also be other important differences between the novels in terms of how they represent political agency, as I’ll suggest below. Yet, in part because the central argument remains unclear, the comparison of the two novels and the importance attached to their similarities and differences is also unclear. For most of the paper they are presented as offering very similar illustrations of the dangers of the Capitalocene and Trumpocene. In which case, why do we need two examples? Their different endings and dystopian/utopian messages are not integrated into the argument. Even the concepts of Capitalocene and Trumpocene seem to be used somewhat interchangeably and don’t play a central role in the argument or analysis. There is a risk in this sort of paper that the conclusions remain at the level of “isn’t this all interesting?” I agree that it certainly is, but would like to push the paper further to say what, precisely, we should take as political implications from these novels, either collectively or comparatively.

 Finally, I’ll offer three comments about areas that seem important but neglected. The first is potentially related to one of the arguments to which the author alludes, that both novels are climate pessimist in that they assume that catastrophic climate change cannot be averted. This confirms the suggestion by Andrew Milner (2020) that adaptation has tended to replace mitigation in climate fiction. Both these novels could be read as examples of political and socio-economic climate adaptation, and the relationship of adaptation to concepts like precarity (and resilience) might be worth exploring. Second, the forms of political agency represented in the novels could be unpacked more, especially in terms of offering insights into how right-wing populism could be challenged (a question the paper raises but does not answer). Several discussions of Robinson’s novel note the complex relationship between individual and collective/networked forms of agency in the story and the revolutionary conclusion (Colebrook, 2019; Cole, 2022). The multi-perspective account could also be reflected upon, especially the significance of ‘the citizen’ narrator whose relationship to ‘the people’ in populist discourses might be unpacked further. Third, there are increasingly important debates about the diversity of perspectives and stories in climate fiction (or the lack thereof). These points have been raised about Robinson’s work in particular (see Hatzisavvidou, 2024; Schneider-Mayerson, 2019) as well as other researchers emphasising the importance of telling different stories about climate politics (Death, 2020; Johns-Putra, 2019; Mitchell and Chaudhury, 2020; Streeby, 2018; Whyte, 2018). In this respect, a stronger justification could be offered for focusing on two stories set in North America, by white male authors with white male leading characters, as well as a deeper analysis of the intersectional inequalities that are represented, reproduced and contested in these stories.

 Overall, this is an enjoying and interesting paper, with some potentially important insights that need a bit more clarity before they can be fully evaluated. I am not convinced, for example, that Robinson’s novel is really about right-wing populism or Trump (although the real estate connection would be interesting to pursue), but I’m also unsure whether this is an important point for the argument that this paper seeks to make. I am persuaded of the importance of stories for envisioning political possibilities and alternatives, and I appreciate that the best stories have an irreducible degree of complexity and ambiguity. As a research paper, however, I wanted this piece to be a bit clearer about what story it is trying to tell.

If any, are all the source data and materials underlying the results available?

No source data required

Is the work original in terms of material and argument?

Yes

Is the work clearly and cogently presented?

Partly

Is the argument persuasive and supported by evidence?

Partly

Does the research article contribute to the cultural, historical, social understanding of the field?

Yes

Does it sufficiently engage with relevant methodologies and secondary literature on the topic?

Partly

Reviewer Expertise:

International Politics, Climate Change, Climate Fiction, Critical Theory

I confirm that I have read this submission and believe that I have an appropriate level of expertise to confirm that it is of an acceptable scientific standard, however I have significant reservations, as outlined above.

## References

[1] : ‘At the heart of human politics’: agency and responsibility in the contemporary climate novel. *Environmental Politics* .2022;31(1) : 10.1080/09644016.2021.1902699 132-151 10.1080/09644016.2021.1902699

[2] : The Future in the Anthropocene: Extinction and the Imagination.2019; 10.1017/9781108505321.017 263-280 10.1017/9781108505321.017

[3] : Envisioning ecopolitical futures: Reading climate fiction as political theory. *Futures* .2024;163: 10.1016/j.futures.2024.103456 10.1016/j.futures.2024.103456

[4] : Eutopia, Dystopia and Climate Change. *Palgrave Macmillan* .2020;

[5] : Worlding beyond ‘the’ ‘end’ of ‘the world’: white apocalyptic visions and BIPOC futurisms. *International Relations* .2020;34(3) : 10.1177/0047117820948936 309-332 10.1177/0047117820948936

[6] : Whose Odds? The Absence of Climate Justice in American Climate Fiction Novels. *ISLE: Interdisciplinary Studies in Literature and Environment* .2019;26(4) : 10.1093/isle/isz081 944-967 10.1093/isle/isz081

[7] : Indigenous science (fiction) for the Anthropocene: Ancestral dystopias and fantasies of climate change crises. *Environment and Planning E: Nature and Space* .2018;1(1-2) : 10.1177/2514848618777621 224-242 10.1177/2514848618777621

[8] : Climate Fiction, Climate Theory: Decolonising Imaginations of Global Futures. *Millennium: Journal of International Studies* .2022;50(2) : 10.1177/03058298211063926 430-455 10.1177/03058298211063926
